# Harmonic Analysis in Phase Space and Finite Weyl–Heisenberg Ensembles

**DOI:** 10.1007/s10955-019-02226-2

**Published:** 2019-01-22

**Authors:** Luís Daniel Abreu, Karlheinz Gröchenig, José Luis Romero

**Affiliations:** 1 Acoustics Research Institute, Austrian Academy of Sciences, Wohllebengasse 12-14, 1040 Vienna, Austria; 2 Faculty of Mathematics, University of Vienna, Oskar-Morgenstern-Platz 1, 1090 Vienna, Austria

**Keywords:** Landau level, Polyanalytic Ginibre ensemble, Hyperuniformity, Weyl-Heisenberg ensemble, Phase-space, Time-frequency analysis

## Abstract

Weyl–Heisenberg ensembles are translation-invariant determinantal point processes on $\mathbb {R}^{2d}$ associated with the Schrödinger representation of the Heisenberg group, and include as examples the Ginibre ensemble and the polyanalytic ensembles, which model the higher Landau levels in physics. We introduce finite versions of the Weyl–Heisenberg ensembles and show that they behave analogously to the finite Ginibre ensembles. More specifically, guided by the observation that the Ginibre ensemble with *N* points is asymptotically close to the restriction of the infinite Ginibre ensemble to the disk of area *N*, we define finite WH ensembles as adequate finite approximations of the restriction of infinite WH ensembles to a given domain $\Omega$. We provide a precise rate for the convergence of the corresponding one-point intensities to the indicator function of $\Omega$, as $\Omega$ is dilated and the process is rescaled proportionally (thermodynamic regime). The construction and analysis rely neither on explicit formulas nor on the asymptotics for orthogonal polynomials, but rather on phase-space methods. Second, we apply our construction to study the pure finite Ginibre-type polyanalytic ensembles, which model finite particle systems in a single Landau level, and are defined in terms of complex Hermite polynomials. On a technical level, we show that finite WH ensembles provide an approximate model for finite polyanalytic Ginibre ensembles, and we quantify the corresponding deviation. By means of this asymptotic description, we derive estimates for the rate of convergence of the one-point intensity of polyanalytic Ginibre ensembles in the thermodynamic limit.

## Introduction

### Weyl–Heisenberg Ensembles

We study the class of determinantal point processes on $\mathbb {R}^{2d}$ whose correlation kernel is given as

1.1 $$\begin{aligned} {K^g}((x,\xi ),(x^{\prime },\xi ^{\prime }))=\int _{\mathbb {R}^{d}}e^{2\pi i(\xi ^{\prime } -\xi )t}g(t-x^\prime ) \overline{g(t-x )}dt \end{aligned}$$

for some non-zero (normalized) function $g\in L^2({\mathbb {R}}^d )$ and $(x,\xi ), (x^{\prime },\xi ^{\prime }) \in \mathbb {R}^{2d}$. These determinantal point processes are called Weyl–Heisenberg ensembles (WH ensembles) and have been introduced recently in [8]. They form a large class of translation-invariant hyperuniform point processes [36, 55, 61].

The prototype of a Weyl–Heisenberg ensemble is the complex Ginibre ensemble. Choosing *g* in (1.1) to be the Gaussian $g(t)=2^{1/4}e^{-\pi t^{2}}$ and writing $z=x+i\xi , z^{\prime }= x^{\prime }+i\xi ^{\prime }$, the resulting kernel is then

1.2 $$\begin{aligned} {K^g}(z,z^{\prime })=e^{i\pi (x^{\prime }\xi ^{\prime }-x\xi )} e^{-\frac{\pi }{2} (\left| z \right| ^{2}+\left| z^{\prime }\right| ^{2})} e^{\pi \overline{z} z^{\prime }}, \qquad z=x+i\xi , \, z^{\prime }= x^{\prime }+i\xi ^{\prime }. \end{aligned}$$

Modulo conjugation with a phase factor, this is essentially the kernel of the *infinite Ginibre ensemble*$K_\infty (z,z^{\prime })= e^{-\frac{\pi }{2} (\left| z\right| ^{2}+\left| z^{\prime }\right| ^{2})}e^{\pi z\overline{z^{\prime }}}$. Another important class of examples arises by choosing *g* to be a Hermite function. In this case one obtains a pure polyanalytic Ginibre ensemble [8, 57], which models the electron density in a single (pure) higher Landau level (see Sect. A.5 for some background).

The Ginibre ensemble with kernel $K_\infty$ arises as limit of corresponding processes with *N* points, whose kernels

1.3 $$\begin{aligned} K_{N}(z,z^{\prime })= e^{-\frac{\pi }{2} (\left| z\right| ^{2}+\left| z^{\prime }\right| ^{2})}\sum _{j=0}^{N-1}\frac{\left( \pi z\overline{ z^{\prime }}\right) ^{j} }{j!}, \end{aligned}$$

are obtained simply by truncating the expansion of the exponential $e^{\pi z \overline{z^{\prime }}}$. It is not obvious how to obtain the analogous finite-dimensional process for a general Weyl–Heisenberg ensemble (1.1), because for most choices of $g\in L^{2}({\mathbb {R}}^d)$ there is no treatable explicit formula available for ${K^g}$. We present a canonical construction of finite Weyl–Heisenberg ensembles and show that they enjoy properties similar to the finite Ginibre ensemble. The construction and analysis is based on spectral theory of Toeplitz-like operators and harmonic analysis of phase space.

The abstract construction is instrumental to study the asymptotic properties of a particularly important class of finite-dimensional determinantal point processes, namely the finite pure polyanalytic Ginibre ensembles, which model the electron density in higher Landau levels. This is an example where the Plancherel–Rotach asymptotics of the basis functions are not available. Moreover, the relevant polynomials do not satisfy the classical three-term recurrence relations which are used in Riemann–Hilbert type methods [25, 27]. We develop a new approach based on spectral methods and harmonic analysis in phase space and show that the finite WH ensembles associated with a Hermite function are asymptotically close to finite polyanalytic ensembles. Thus, our analysis of the finite polyanalytic ensembles has two steps: (i) the abstract construction of finite WH ensembles and their thermodynamic limits; (ii) the comparison of the finite WH ensembles associated with Hermite functions and the finite pure polyanalytic ensembles.

### Planar Hermite Ensembles

The complex Hermite polynomials are given by

1.4 $$\begin{aligned} H_{j,r}(z,\overline{z})=\left\{ \begin{array}{ll} {\sqrt{\frac{r!}{j!}}\pi ^{\frac{j-r}{2}}z^{j-r}L_{r}^{j-r}\left( \pi \left| z\right| ^{2}\right) , \qquad j>r \ge 0,} \\ {\left( -1\right) ^{r-j}\sqrt{\frac{j!}{r!}}\pi ^{\frac{r-j}{2}}\overline{ z }^{r-j}L_{j}^{r-j}\left( \pi \left| z\right| ^{2}\right) , \qquad 0 \le j\le r}, \end{array} \right. \end{aligned}$$

where $L_{r}^\alpha$ denotes the Laguerre polynomial

1.5 $$\begin{aligned} L_{j}^{\alpha }(x)=\sum \limits _{i=0}^{j}(-1)^{i}\left( {\begin{array}{c}j+\alpha \\ j-i\end{array}}\right) \frac{ x^{i}}{i!}, \qquad x\in {\mathbb {R}}, \qquad j \ge 0, j+\alpha \ge 0. \end{aligned}$$

Complex Hermite polynomials satisfy the doubly-indexed orthogonality relation

$$\begin{aligned} \int _{\mathbb {C}}H_{j,r}(z,\overline{z})\overline{H_{j\prime ,r\prime }(z, \overline{z})}e^{-\pi \left| z\right| ^{2}}dz={\delta }_{jj\prime }{ \delta }_{rr\prime }, \end{aligned}$$

and form an orthonormal basis of $L^{2}\left( \mathbb {C}, e^{-\pi \left| z\right| ^{2}}\right)$ [4].^1^

The complex Hermite polynomials form a complete set of eigenfunctions of the Landau operator

1.6 $$\begin{aligned} L_{z}:=-\partial _{z}\partial _{\overline{z}}+\pi \overline{z}\partial _{ \overline{z}} \end{aligned}$$

acting on the Hilbert space $L^{2}( \mathbb {C}, e^{-\pi \left| z\right| ^{2}})$. The Landau operator is the Schrödinger operator that models the behavior of an electron in $\mathbb {R}^2$ in a constant magnetic field perpendicular to the $\mathbb {C}$-plane. The spectrum of $L_{z}$, i.e., the set of possible energy levels, is given by $\sigma (L_{z})=\{r\pi :r=0,1,2,\ldots \}$ and the eigenspace associated with the eigenvalue $r\pi$ is called the * Landau level of order r*. For the minimal energy $r=0$, i.e., the ground state, the eigenspace is the classical Fock space, for $r>0$, the eigenspaces are spanned by the orthonormal basis $\{ H_{j,r}: j\in {\mathbb {N}}\}$. The Landau levels are key for the mathematical formulation of the integer quantum Hall effect discovered by von Klitzing [64].

We will consider a variety of ensembles associated with the complex Hermite polynomials.

#### Definition 1.1

Let $J \subseteq {\mathbb {N}}_{0}\times {\mathbb {N}}_{0}$. The planar Hermite ensemble based on *J* is the determinantal point process with the correlation kernel

1.7 $$\begin{aligned} K(z,z^{\prime })= e^{-\frac{\pi }{2} ({\left| z\right| ^{2}+\left| z^{\prime }\right| ^{2}})}\sum _{j,r\in J}H_{j,r} \left( z,\overline{z} \right) \overline{ H_{j,r} \left( z^{\prime },\overline{z^{\prime }} \right) } . \end{aligned}$$

Complex Hermite polynomials are an example of *polyanalytic functions*—that is, polynomials in $\overline{z}$ with analytic coefficients (see Sect. A.4). While most classes of orthogonal polynomials satisfy a three-term recurrence relation—which puts them in the scope of Riemann-Hilbert type techniques [25, 27]—the complex Hermite polynomials satisfy instead a system of doubly-indexed recurrence relations [34, 45].

Several important determinantal point processes arise as special cases of (1.7). First, since $H_{j,0}(z,\overline{z} )=(\pi ^{j}/j!)^{\frac{1}{2}}z^{j}$, the set $J=\{0,\ldots ,N-1\}\times \{0\}$ in (1.7) leads to the kernel of the Ginibre ensemble (1.3). A second important example arises for $J := \{(j,r): 0 \le j \le n-1, r=m-n+j\}$ with $n,m \in \mathbb {N}$. The corresponding one-point intensity is a radial version of the marginal probability density function of the unordered eigenvalues of a complex Gaussian Wishart matrix after the change of variables $t \rightarrow \pi \left| z \right| ^2$, see, e.g. [62, Theorem 2.17]. Thirdly, choosing $J=\{0,\ldots ,N-1\}\times \{0,\ldots ,q-1\}$ one obtains the polyanalytic Ginibre ensemble introduced by Haimi and Hedenmalm [40]. The polyanalytic Ginibre ensemble gives the probability distribution of a system composed by several Landau levels. The case of more general interaction potentials has been investigated in [40, 41], by considering polyanalytic Ginibre ensembles with general weights. These investigations parallel the ones of weighted Ginibre ensembles [9–11].

We are particularly interested in finite versions of the infinite pure polyanalytic ensembles defined by Shirai [57]. The infinite ensembles are defined by the reproducing kernels of an eigenspace of the Landau operator (1.6) which is given by

$$\begin{aligned} K_{r}(z,z^{\prime })=L_{r}^{0}(\pi \left| z-z^{\prime }\right| ^{2})e^{\pi z\overline{w}-\tfrac{\pi }{2}(\left| z\right| ^{2}+\left| z^{\prime }\right| ^{2})}=e^{-\frac{\pi }{2}(\left| z\right| ^{2}+\left| z^{\prime }\right| ^{2})}\sum _{j=0}^{\infty }H_{j,r}\left( z,\overline{z}\right) \overline{H_{j,r}\left( z^{\prime }, \overline{z^{\prime }}\right) }\text {.} \end{aligned}$$

Here the second identity follows from the fact that $\left\{ H_{j,r}\left( z,\overline{z}\right) \right\} _{j\in \mathbb {N} }$ spans the *rth* eigenspace of the Landau operator. The corresponding finite pure polyanalytic ensembles can now be defined as planar Hermite ensembles with $J=\{0,\ldots ,N-1\}\times \{r\}$. In analogy to (1.3), the finite *(r, N)-pure polyanalytic ensemble* is the determinantal point process with correlation kernel

1.8 $$\begin{aligned} K_{r,N}(z,z^{\prime })= e^{-\frac{\pi }{2} (\left| z\right| ^{2}+\left| z^{\prime }\right| ^{2})} \sum _{j=0}^{N-1} H_{j,r} \left( z,\overline{z} \right) \overline{H_{j,r}\left( z^{\prime },\overline{z^{\prime }}\right) }. \end{aligned}$$

While pure polyanalytic ensembles describe individual Landau levels, their finite counterparts model a finite number of particles confined to a certain disk (for example, as the result of a radial potential). In this article, we prove the following theorem, which supports this interpretation, and provides a rate of convergence for the one-point intensity related to each Landau level.

#### Theorem 1.2

Let $\rho _{r,N}(z)=K_{r,N}(z,z)$ be the one-point intensity of the finite (*r*, *N*)-pure polyanalytic Ginibre ensemble. Then, for each $r>0$,

1.9 $$\begin{aligned} \rho _{r,N} \Big (\sqrt{\tfrac{N}{\pi }} \, \cdot \Big )\longrightarrow 1_{ \mathbb {D}}, \end{aligned}$$

in $L^{1}({\mathbb {R}^{2}})$, as $N\longrightarrow +\infty$. Moreover,

1.10 $$\begin{aligned} \bigl || \rho _{r,N}-1_{\mathbb {D}_{\sqrt{N/\pi }}} \bigr || _{1}\le C_{r} \sqrt{N}. \end{aligned}$$

The convergence rate in Theorem 1.2 is independent of the energy level *r* of the Landau operator. It is known to be sharp for the first Landau level $$r=0$$, and we believe that (1.10) is also sharp for all Landau levels $$r\in {\mathbb {N}}$$.^2^$$^{,}$$^3^

In statistical terms, (1.10) means that the number of points of the (*r*, *N*)-pure polyanalytic Ginibre ensemble that belong to a certain domain $A \subseteq \mathbb {C}$, $n_{r,N}(A)$, satisfies

1.11 $$\begin{aligned} \mathbb {E}\{ n_{r,N}(A) \} = \left| \mathbb {D}_{\sqrt{N/\pi }} \cap A \right| + \mathcal {O}\big (\sqrt{N}\big ). \end{aligned}$$

Theorem 1.2 supports and validates the interpretation of finite pure polyanalytic ensembles as models for *N* particles confined to a disk by giving asymptotics for the first order statistics (1.11) that indeed show concentration on the disk area *N*, up to an error comparable to the perimeter of that disk. In addition, (1.11) implies that, after proper rescaling, the particles are, in expectation, asymptotically equidistributed on the disk. This statistical description is consistent with the notion of a *filling factor* of each Landau level—that is, a certain limit to the number of particles that each level can accommodate. The incremental saturation of each individual Landau level, corresponding to incremental energy levels, is part of the mathematical description of the integer quantum Hall effect discovered by von Klitzing [64]. (The integer quantum Hall effect is not to be confused with the fractional quantum Hall effect, whose mathematical formulation is related to the Laughlin’s wave function [48] and the so-called beta-ensembles [18, 19].)

As a first step towards a description of finite pure polyanalytic ensembles, we introduce a general construction of finite versions of Weyl–Heisenberg ensembles that may be of independent interest.

### Finite Weyl–Heisenberg Ensembles

The construction of finite WH ensembles relies on methods from harmonic analysis on phase space [32, 33], and on the spectral analysis of phase-space Toeplitz operators. Write $z=(x,\xi ) \in {{\mathbb {R}}^{2d}}, z^{\prime }= (x^{\prime },\xi ^{\prime }) \in {{\mathbb {R}}^{2d}}$ for a point in phase space and

1.12 $$\begin{aligned} \pi (z)f(t):=e^{2\pi i\xi t}f(t-x) \end{aligned}$$

for the phase-space shift by *z*. Then the kernel in (1.1) is given by

1.13 $$\begin{aligned} {K^g}(z,z^{\prime }) = \langle \pi (z^{\prime })g, \pi (z)g\rangle . \end{aligned}$$

Let us now describe the construction of the finite point processes associated with the kernel ${K^g}$. For normalized $g\in L^2({\mathbb {R}}^d )$, $\Vert g\Vert _2 = 1$, the integral operator with kernel ${K^g}$, i.e., $F \mapsto \int _{\mathbb {R}^{2d}} {K^g}(z,z^{\prime }) F(z^{\prime }) dz^{\prime }$, is an orthogonal projection (see for example [32, Chapter 1], [38, Chapter 9]). Consequently, the range of this projection is a *reproducing kernel Hilbert space*$\mathcal {V}_g \subseteq L^2({{\mathbb {R}}^{2d}} )$ with the explicit description

$$\begin{aligned} \mathcal {V}_g =\big \{F \in L^2({{\mathbb {R}}^{2d}} ): F(z) = \langle f, \pi (z) g\rangle , \text{ for } f \in L^2({\mathbb {R}}^d)\,\big \} \subseteq {L^{2}({\mathbb {R} ^{2d}})}. \end{aligned}$$

Thus every $F\in \mathcal {V} _g$ is a phase-space representation of a function *f* defined on the configuration space $\mathbb {R}^d$.

*Step 1: Concentration as a smooth restriction* Let $\mathcal {X}^g$ be a WH ensemble (with correlation kernel ${K^g}$) and let $\Omega \subseteq {\mathbb {R}^{2d}}$ be a measurable set. The restriction of $\mathcal {X}^g$ to $\Omega$ is a determinantal point process (DPP) $\mathcal {X}^g_{|\Omega }$ with correlation kernel

1.14 $$\begin{aligned} {K^g}_{|\Omega }(z,z^{\prime }) = 1_\Omega (z) {K^g}(z,z^{\prime }) 1_\Omega (z^{\prime }). \end{aligned}$$

An expansion of the kernel ${K^g}_{|\Omega }$ can be obtained as follows. We consider the *Toeplitz operator* on $\mathcal {V}_g$ defined by

1.15 $$\begin{aligned} M_{\Omega }^{g}F(z) =\int _{\Omega }F(z^{\prime \prime }){K^g}(z,z^{\prime \prime })\,dz^{\prime \prime } \, . \end{aligned}$$

Since $F(z^{\prime \prime } ) = \int _{{{\mathbb {R}}^{2d}}} F(z^\prime ) K^g(z^{\prime \prime },z^\prime ) \, dz^\prime$ for $F \in \mathcal {V}_g$, $M_{\Omega }^{g}$ can be expressed as an integral operator

1.16 $$\begin{aligned} M_{\Omega }^{g}F(z)&=\int _{\mathbb {R}^{2d}}F(z^{\prime \prime }) \, 1_\Omega (z^{\prime \prime }){K^g}(z,z^{\prime \prime })\,dz^{\prime \prime } \end{aligned}$$

1.17 $$\begin{aligned}&= \int _{{\mathbb {R}^{2d}}} F(z^{\prime }) \left[ \int _{{\mathbb {R}^{2d}}} {K^g} (z,z^{\prime \prime }) 1_\Omega (z^{\prime \prime }) {K^g} (z^{\prime \prime },z^{\prime }) \, dz^{\prime \prime }\right] \, dz^{\prime }. \end{aligned}$$

By definition (1.15), $M_{\Omega }^{g}$ acts on a function $F \in \mathcal {V}_g$ by multiplication by $1_\Omega$, followed by projection onto $\mathcal {V}_g$. On the other hand, if $F \in \mathcal {V}_g^\perp$, then the expression in (1.17) vanishes. Thus, the formula in (1.17) defines the extension of $M_{\Omega }^{g}$ to $L^2(\mathbb {R}^{2d})$ that is 0 on $L^2(\mathbb {R}^{2d}) \ominus \mathcal {V}_g$. For $\Omega \subseteq {{\mathbb {R}}^{2d}}$ of finite measure, $M^g_\Omega$ is a compact positive (self-adjoint) operator on $L^2({{\mathbb {R}}^{2d}} )$; see for example [21, 54]. By the spectral theorem, $M^g_\Omega$ is diagonalized by an orthonormal set $\{p_{g,j}^{\Omega }: j \in {\mathbb {N}}\} \subseteq \mathcal {V}_g$ of eigenfunctions, with corresponding eigenvalues $\lambda _j = \lambda _{j}^{\Omega }$ (ordered non-increasingly):

1.18 $$\begin{aligned} M_{\Omega }^{g} = \sum _{j \ge 1} \lambda _{j}^{\Omega } \, p_{g,j}^{\Omega } \otimes p_{g,j}^{\Omega }. \end{aligned}$$

The key property is that the eigenfunctions $p_{g,j}^{\Omega }$ are * doubly-orthogonal*: since

$$\begin{aligned} \left<M_{\Omega }^{g} F,F\right> = \int _\Omega \left| F(z) \right| ^2 dz, \qquad F \in L^2(\mathbb {R}^{2d}), \end{aligned}$$

it follows that

$$\begin{aligned} \left<p_{g,j}^{\Omega },p_{g,j^{\prime }}^{\Omega }\right> _{L^2(\Omega )} =\left<M_{\Omega }^{g} p_{g,j}^{\Omega },p_{g,j^{\prime }}^{\Omega }\right> _{L^2({\mathbb {R}^{2d}})} = \lambda _{j}^{\Omega } \delta _{j,j^{\prime }}, \end{aligned}$$

and consequently the restricted kernel has the orthogonal expansion

1.19 $$\begin{aligned} {K^g}_{|\Omega }(z,z^{\prime }) = \sum _{j \ge 1} \left( p_{g,j}^{\Omega }(z)1_\Omega (z) \right) \cdot \left( \overline{ p_{g,j}^{\Omega }(z^{\prime })}1_\Omega (z^{\prime }) \right) ; \end{aligned}$$

see Sect. 6.1 for details. Note that in (1.19), the functions $p_{g,j}^{\Omega }(z)1_\Omega (z)$ are not normalized. In fact,

1.20 $$\begin{aligned} \int _\Omega \left| p_{g,j}^{\Omega }(z) \right| ^2 dz = \lambda _{j}^{\Omega }. \end{aligned}$$

Thus, while in (1.19) the basis functions are restricted to the domain $\Omega$, the expansion of the Toeplitz operator (1.18) involves the non-truncated functions $p_{g,j}^{\Omega }(z)$ weighted by the measure of their concentration on $\Omega$ (1.20). We call the DPP with correlation kernel corresponding to (1.17) the *concentration* of the full WH ensemble to $\Omega$ and denote it by $\mathcal {X}^{g,\mathrm {con}}_{\Omega }$. This process is thus a smoother variant of the restricted process $\mathcal {X}^{g}_{|\Omega }$, because it involves the (smooth) functions $p_{g,j}^{\Omega }(z)$ instead of their truncations $p_{g,j}^{\Omega }(z) 1_\Omega (z)$, which may have discontinuities along $\partial \Omega$. The construction of DPPs from the spectrum of self-adjoint operators has been suggested in [16, 17] as an analogue of the construction of DPPs from the spectral measure of a group. In a related work [52], a combination of methods from operator theory and representation theory has been used to show that a DPP is the spectral measure for an explicit commutative group of Gaussian operators in the fermionic Fock space.

*Step 2: Spectral truncation* Since $\left<M_{\Omega }^{g} F,F\right> = \int _\Omega \left| F \right| ^2$, by the min-max principle,

1.21 $$\begin{aligned} \lambda ^\Omega _j = \max \left\{ \int _\Omega \left| F(z) \right| ^2 dz: \left\| F\right\| _2=1, F \in \mathcal {V}_g, F \perp p_{g,1}^{\Omega }, \ldots , p_{g,j-1}^{\Omega } \right\} . \end{aligned}$$

Thus, the eigenvalues $\lambda _{j}^{\Omega }$ describe the best possible simultaneous phase-space concentration of waveforms within $\Omega$. In particular, (1.21) implies that

$$\begin{aligned} 0 \le \lambda ^\Omega _j \le 1, \qquad j \ge 1. \end{aligned}$$

It is well-known that there are $\approx \left| \Omega \right|$ eigenvalues $\lambda _{j}^{\Omega }$ that are close to 1. As a precise statement we cite the following *Weyl-type law*: for any $\delta \in (0,1)$,

1.22 $$\begin{aligned} \left| \#\{j:\lambda _{j}^{\Omega }>1-\delta \}-\left| \Omega \right| \right| \le \max \left\{ \frac{1}{\delta }, \frac{1}{1-\delta } \right\} C_{g} \left| \partial \Omega \right| _{2d-1}, \end{aligned}$$

where $\left| \partial \Omega \right| _{2d-1}$ is the perimeter of $\Omega$ (the surface measure of its boundary), and $C_{g}$ is a constant depending explicitly on *g*. See for instance [6, Proposition  3.4] or [24]. The dependence of the constant $C_g$ on *g* is made explicit below in (1.27).

We now look into the concentrated process $\mathcal {X}^{g,\mathrm {con} }_{\Omega }$ introduced in Step 1. The Toeplitz operator $M^g_\Omega$ is not a projection. However, the corresponding DPP can be realized as a random mixture of DPP’s associated with projection kernels [44, Theorem 4.5.3]. Indeed, if $I_j \sim \text{ Bernoulli }(\lambda _{j}^{\Omega })$ are independent (taking the value 1 or 0 with probabilities $\lambda _{j}^{\Omega }$ and $1-\lambda _{j}^{\Omega }$ respectively), then $\mathcal {X}^{g,\mathrm {con}}_{\Omega }$ is generated by the kernel corresponding to the random operator

1.23 $$\begin{aligned} M_{\Omega }^{g, \mathrm {ran}} = \sum _{j \ge 1} I_j \cdot p_{g,j}^{\Omega } \otimes p_{g,j}^{\Omega }. \end{aligned}$$

Precisely, this means that one first chooses a realization of the $I_j$’s and then a realization of the DPP with the kernel above. Because of (1.22), the first eigenvalues $\lambda _j$ are close to 1 and thus the corresponding $I_j$ will most likely be 1. Similarly, for $j \gg \left| \Omega \right|$, the corresponding $I_j$ will most likely be 0. As a finite-dimensional model for WH ensembles, we propose replacing the random Bernoulli mixing coefficients with

1.24 $$\begin{aligned} \left\{ \begin{aligned}&1,&\quad {\text { for }} j \le \left| \Omega \right| , \\&0,&\quad {\text { for }} j > \left| \Omega \right| . \end{aligned} \right. \end{aligned}$$

#### Definition 1.3

Let $g \in L^2({\mathbb {R}^d})$ be of norm 1—called *the window function*, let $\Omega \subseteq {\mathbb {R}^{2d}}$ with non-empty interior and finite measure and perimeter, and let $N_{\Omega }=\left\lceil \left| \Omega \right| \right\rceil$ the least integer greater than or equal to the Lebesgue measure of $\Omega$. The *finite Weyl–Heisenberg ensemble* is the determinantal point process $\mathcal {X}^g_\Omega$ with correlation kernel^4^

$$\begin{aligned} K_{g,\Omega }(z,z^{\prime })=\sum _{j=1}^{N_{\Omega }}p_{g,j}^{\Omega }(z) \overline{p_{g,j}^{\Omega }(z^{\prime })}. \end{aligned}$$

To illustrate the construction, consider $$g(t) = 2^{1/4} e^{-\pi t^2}$$ and $$ \Omega = D_R = \{ z\in {\mathbb {C}}: |z| \le R\} $$. The eigenfunctions of $$ M_{D_R}^g$$ are explicitly given as $$p_{g,j}^{D_R}(\overline{z}) = e^{\pi i x \xi } (\pi ^{j}/j!)^{\frac{1}{2}}z^{j} e^{-\pi |z|^2/2}$$, $$z=x+i\xi $$. They are independent of the radius *R* of the disk, and choosing *R* such that $$ \left| D_R \right| =N$$, the corresponding finite WH ensemble is precisely the finite Ginibre ensemble given by (1.3). This well known fact also follows as a special case from Corollary 4.6.

### Scaled Limits and Rates of Convergence

We now discuss how finite WH ensembles behave when the number of points tends to infinity. Let

$$\begin{aligned} \rho _{g,\Omega }(z) = K_{g,\Omega } (z,z) = \sum _{j=1}^{N_{\Omega }}|p_{g,j}^{\Omega }(z) |^2 \end{aligned}$$

be the one-point intensity of a finite Weyl–Heisenberg ensemble, so that

$$\begin{aligned} \int _D \rho _{g,\Omega } (z) dz = \mathbb {E}\left[ \mathcal {X}^g_\Omega (D) \right] \, \end{aligned}$$

is the expected number of points to be found in $D \subseteq {{\mathbb {R}}^{2d}}$ (see Sect. 1). The following describes the scaled limit of the one-point intensities.

#### Theorem 1.4

Let $\Omega \subset {\mathbb {R}^{2d}}$ be compact. Then the 1 -point intensity of the finite Weyl–Heisenberg ensemble satisfies

1.25 $$\begin{aligned} \rho _{g,m\Omega }(m\cdot )\longrightarrow 1_{\Omega }, \end{aligned}$$

in $$L^{1}({\mathbb {R}^{2d}})$$, as $$m\longrightarrow +\infty $$.

In statistical terms, the convergence in Theorem 1.4 means that, as $m \longrightarrow \infty$,

1.26 $$\begin{aligned} \begin{aligned} \tfrac{1}{m^{2d}} \mathbb {E}\left[ \mathcal {X}^g_{m\Omega }(mD) \right]&= \tfrac{1}{m^{2d}} \int _{mD} \rho _{g,m\Omega } (z) dz = \int _{D} \rho _{g,m\Omega } (mz) dz \\&\longrightarrow \int _{D} 1_\Omega (z) dz = \left| D \cap \Omega \right| . \end{aligned} \end{aligned}$$

Theorem 1.4 follows immediately from [6, Theorem 1.3], once the one-point intensity $\rho _{g,\Omega }$ is recognized as the accumulated spectrogram studied in [6, Definition 1.2]. We make a few remarks as a companion to the illustrations in Figs. 2 and 3.

(i) When $g(t) = 2^{1/4} e^{-\pi t^2}$ and $\Omega$ is a disk of area *N*, Theorem 1.4 follows from the circular law of the Ginibre ensemble.
(ii) The asymptotics are not restricted to disks, but hold for arbitrary sets $\Omega$ with finite measure and also hold in arbitrary dimension, not just for planar determinantal point processes.
(iii) The limit distribution in (1.25) is independent of the parameterizing function *g*. This can be seen as an another instance of a universality phenomenon [26, 50, 59].

**Fig. 1:**
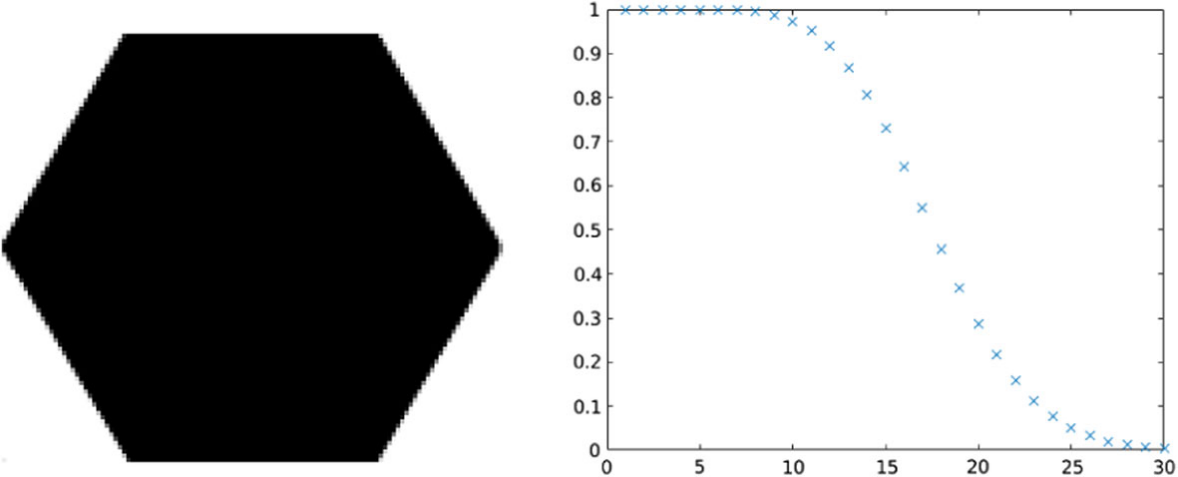
A plot of the eigenvalues of the Toeplitz operator $M^g_\Omega$, with *g* a Gaussian window and $\Omega$ of area $\approx$ 18

**Fig. 2:**
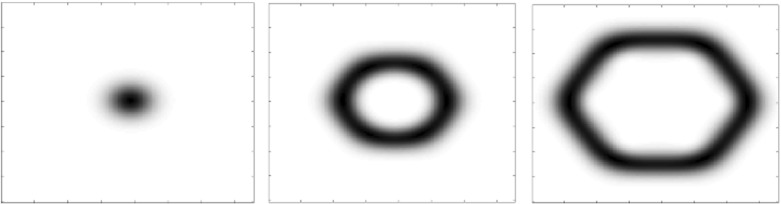
The eigenfunctions # 1, 7, 18 corresponding to the operator in Fig. 1

**Fig. 3:**
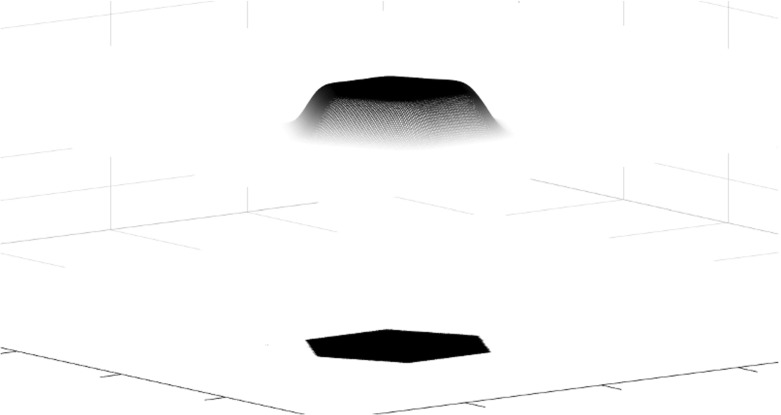
The one-point intensity of a WH ensemble plotted over the domain in Fig. 1

In view of Theorem 1.4 we will quantify the deviation of the finite WH ensemble from its limit distribution in the $L^1$-norm, using the results in [7], where the sharp version of the main result in [6] has been obtained.

#### Theorem 1.5

Let $\rho _{g,\Omega }$ be the one-point intensity of the finite Weyl–Heisenberg ensemble. Assume that *g* satisfies the condition

1.27 $$\begin{aligned} ||g||_{M^{*}}^{2}:=\int _{\mathbb {R}^{2d}}\left| z\right| \left| \langle g, \pi (z)g\rangle \right| ^{2}dz<+\infty . \end{aligned}$$

If $\Omega$ has finite perimeter and $\left| \partial \Omega \right| _{2d-1} \ge 1$, then

1.28 $$\begin{aligned} ||\rho _{g,\Omega }-1_{\Omega }||_{1}\le C_{g} \left| \partial \Omega \right| _{2d-1} \end{aligned}$$

with a constant depending only on $\Vert g\Vert _{M^*}$.

The condition on the window *g* in (1.27) amounts to mild decay in the time and frequency variables, and is satisfied by every Schwartz function. See Sects. 5.1 and A.3 for a discussion on closely-related function classes. The error rate in Theorem 1.5 is sharp—see [7, Theorem 1.6]. Intuitively, in (1.28) we compare the continuous function $\rho _{g,\Omega }$ with the characteristic function $1_{\Omega }$. Thus, along every point of the boundary of $\Omega$ (of surface measure $\left| \partial \Omega \right| _{2d-1}$) we accumulate a pointwise error of $\mathcal {O} (1)$, leading to a total $L^1$-error at least of order $\left| \partial \Omega \right| _{2d-1}$ .

### Approximation of Finite Polyanalytic Ensembles by WH Ensembles

The second ingredient towards the proof of Theorem 1.2 is a comparison result that bounds the deviation between finite pure polyanalytic ensembles and finite WH ensembles with Hermite window functions. Before stating the result, some preparation is required. We consider the following transformation, which is usually called a gauge transformation, and the change of variables $f^*(z):=f( \overline{z})$, $z \in {\mathbb {C}}^d$. Given an operator $T: L^2({\mathbb {R}^{2d}}) \rightarrow L^2( {\mathbb {R}^{2d}})$ we denote:

1.29 $$\begin{aligned} \left[ {\widetilde{T}} f \right] ^* := \overline{m} \, T(f^* \, m), \qquad m(x,\xi ) := e^{-\pi i x \xi }. \end{aligned}$$

Hence, if *T* has the integral kernel *K*, then ${\widetilde{T}}$ has the integral kernel

1.30 $$\begin{aligned} {\widetilde{K}}(z,z^{\prime })=e^{ \pi i (x^{\prime }\xi ^{\prime }- x \xi )} K \left( \overline{z},\overline{z^{\prime }} \right) , \qquad z=x+ i \xi ,\, z^{\prime }=x^{\prime }+i\xi ^{\prime }. \end{aligned}$$

(See Sect. 1 for details). We call the operation $K \mapsto {\widetilde{K}}$ a renormalization of the kernel *K*. With this notation, if ${K^g}$ is the kernel in (1.2) and *g* is the Gaussian window, then $\widetilde{K}_g$ is the kernel of the infinite Ginibre ensemble. In addition, the DPP’s on ${\mathbb {C}}^d$ associated with the kernels *K* and $\widetilde{K}$ are related by the transformation $z \mapsto \overline{z}$. Now, let the window *g* be a Hermite function

1.31 $$\begin{aligned} h_{r}(t) = \frac{2^{1/4}}{\sqrt{r!}}\left( \frac{-1}{2\sqrt{\pi }}\right) ^r e^{\pi t^2} \frac{d^r}{dt^r}\left( e^{-2\pi t^2}\right) , \qquad r \ge 0. \end{aligned}$$

The corresponding kernel $K_{h_r}$ describes (after the renormalization above) the orthogonal projection onto the Bargmann-Fock space of pure polyanalytic functions of type *r* (see Sect. A.4).

Let us consider a Toeplitz operator on $$L^2(\mathbb {R}^2)$$ with a circular domain $$\Omega = D_R$$. By means of an argument based on phase-space symmetries (more precisely, the symplectic covariance of Weyl’s quantization) we show in Sect. 4 that the eigenfunctions $$ \{{\widetilde{p}}_{h_r,j}^{D_R}: j \ge 1\}$$ of $$\widetilde{M}^{h_r}_{D_R}$$ are the normalized complex Hermite polynomials $$H_{j,r}(z,{\bar{z}}) e^{-\frac{\pi }{2} |z|^2}$$. In particular, as with the Ginibre ensemble, the eigenfunctions are independent of the radius *R*. Choosing *R* such that $$ N_{D_R} = N$$, and recalling that we order the eigenvalues of $$M_{D_R}^{h_r}$$ by magnitude, we obtain a map $$\sigma : {\mathbb {N}}_0 \rightarrow {\mathbb {N}}_0$$, such that

$$\begin{aligned} {\widetilde{p}}_{h_r,j}^{D_R}=H_{\sigma (j),r}(z,{\bar{z}}) e^{-\frac{\pi }{2} |z|^2}. \end{aligned}$$

Thus, the finite WH ensemble associated with $h_r$ and $D_R$ is a planar Hermite ensemble, with correlation kernel

1.32 $$\begin{aligned} {\widetilde{K}}_{h_r,D_{R}}(z,z^{\prime })=e^{ -\frac{\pi }{2} (\left| z\right| ^{2}+\left| z^{\prime }\right| ^{2})}\sum _{j=1} ^{N_{D_{R}} } H_{\sigma (j),r}(z,\overline{z}) \overline{H_{\sigma (j),r}(z^{\prime }, \overline{z^{\prime }})}. \end{aligned}$$

Comparing the correlation kernels of the finite pure polyanalytic ensemble (1.8) with the finite (renormalized) WH ensemble with a Hermite window (1.32), we see that in each case different subsets of the complex Hermite basis intervene: in one case functions are ordered according to their Hermite index, while in the other they are ordered according to the magnitude of their eigenvalues.

**Fig. 4:**
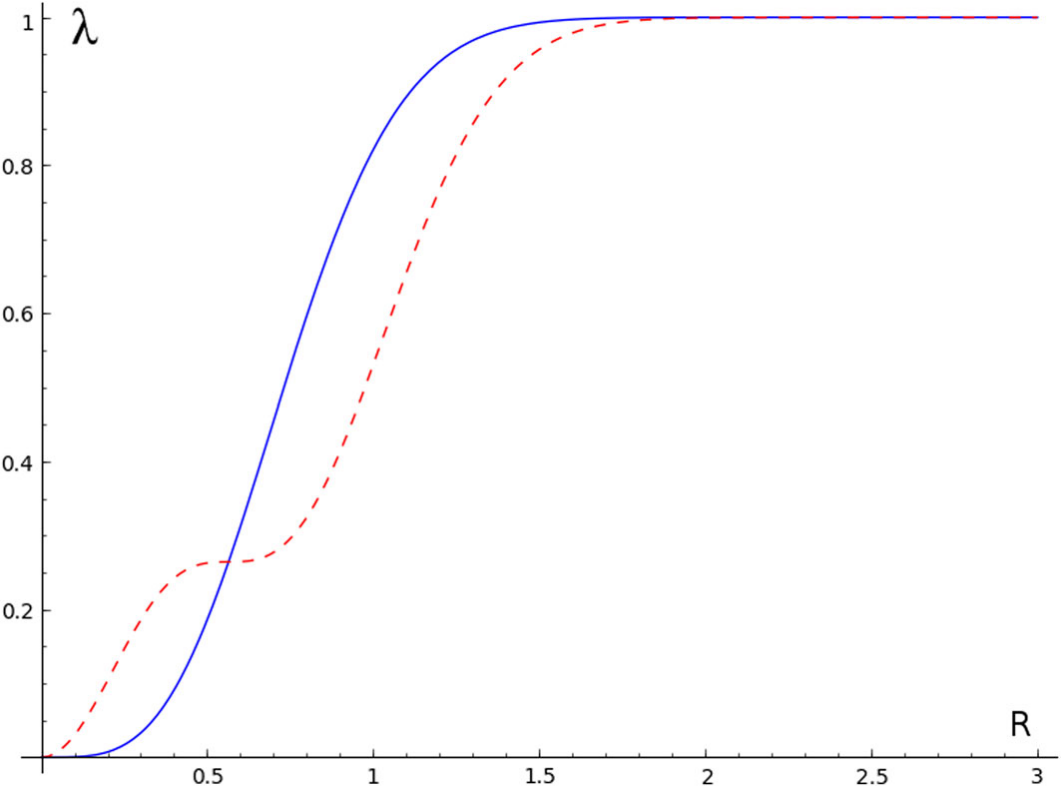
A plot of the eigenvalues $\lambda =\widetilde{M} ^{h_1}_{D_R} \left( H_{j,1}(z,\overline{z}) e^{-\tfrac{\pi }{2}\left| z \right| ^2} \right)$, as a function of *R*, corresponding to $j=0$ (blue, solid) and $j=1$ (red, dashed) (Color figure online)

Figure 4 shows the eigenvalues of $$\widetilde{M}^{h_1}_{D_R}$$, as a function of *R*, corresponding to the eigenfunctions $$H_{0,1}(z, \overline{z}) e^{-\tfrac{\pi }{2}\left| z \right| ^2}$$ and $$H_{1,1}(z, \overline{z}) e^{-\tfrac{\pi }{2}\left| z \right| ^2}$$. For small values of $$ R>0$$, the eigenvalue corresponding to $$H_{1,1}$$ is bigger than the one corresponding to $$H_{1,0}$$, and thus for small *N*, the kernels in (1.8) and (1.32) do not coincide. The following result shows that this difference is asymptotically negligible.

#### Theorem 1.6

Let $N \in {\mathbb {N}}$ and $R>0$ be such that $N_{D_R}= \lceil \left| D_R \right| \rceil =N$. Let $K_{h_r, D_{R}}$ be the correlation kernel of the finite Weyl–Heisenberg ensemble associated with the Hermite window $h_r$ and the disk $D_{R}$, and $K_{r,N}$ the correlation kernel of the (*r*, *N*)-pure polyanalytic ensemble given by (1.8). Then

$$\begin{aligned} \bigl ||{\widetilde{K}}_{h_r, D_{R}} - K_{r,N}\bigr ||_{S^1} \lesssim \left| \partial D_{R} \right| _{1} \asymp \sqrt{N}, \end{aligned}$$

where $\left\| \cdot \right\| _{S^1}$ denotes the trace-norm of the corresponding integral operators.

Since $\left\| K_{h_r, D_{R}}\right\| _{S^1} = \left\| K_{r,N}\right\| _{S^1} = N$, the finite pure polyanalytic ensemble—defined by a lexicographic criterion—is asymptotically equivalent to a finite WH ensemble - defined by optimizing phase-space concentration. To derive Theorem 1.6, we resort to methods from harmonic analysis on phase space. More precisely, we will use Weyl’s correspondence and account for the difference between (1.32) and (1.8) as the error introduced by using two different variants of Berezin’s quantization rule (anti-Wick calculus).

Finally, Theorem 1.2 follows by combining the comparison result in Theorem 1.6 with the asymptotics in Theorem 1.5 applied to Hermite windows—see Sect. 5.4. This argument is reminiscent of Lubinsky’s localization principle [50] that concerns deviations between kernels of orthogonal polynomials. In the present context, the difference between the two kernels does not stem from an order relation between two measures, but from a permutation of the basis functions.

### Simultaneous Observability

The independence of the eigenfunctions of $M_{D_R}^{h_r}$ of the radius *R* yields another property of the (finite and infinite) *r*-pure polyanalytic ensembles.

#### Theorem 1.7

The restrictions $\{ p_{h_r,j} \big | _{D_R} : j\in {\mathbb {N}}\}$ are orthogonal on $L^2(D_R)$ for all $R>0$. In the terminology of determinantal point processes this means that the family of disks $\{D_R: R>0\}$ is simultaneously observable for all *r*-pure polyanalytic ensembles.

This recovers and slightly extends a result of Shirai [57]. As an application, we obtain an extension of Kostlan’s theorem [47] on the absolute values of the points of the Ginibre ensemble of dimension *N*.

#### Theorem 1.8

The set of absolute values of the points distributed according to the *r*-pure polyanalytic Ginibre ensemble has the same distribution as $\{Y_{1,r},\ldots ,Y_{n,r}\}$, where the $Y_{j}$’s are independent and have density

$$\begin{aligned} f_{Y_{j}}(x):=2 \frac{\pi ^{j-r+1} r!}{j!} x^{2(j-r)+1}\left[ L_{r}^{j-r}(\pi x^2)\right] ^{2}e^{-\pi x^2}, \end{aligned}$$

where $L_{j}^{\alpha }$ are the Laguerre polynomials of (1.5). (Hence, $Y^2_j$ is distributed according to a generalized Gamma function with density $f_{Y_j^2}(x) = \frac{\pi ^{j-r+1} r!}{j!} x^{j-r}\left[ L_{r}^{j-r}(\pi x)\right] ^{2}e^{-\pi x}$).

### Organization

Section 2 presents tools from phase-space analysis, including the short-time Fourier transform and Weyl’s correspondence. Section 3 studies finite WH ensembles and more technical variants required for the identification of finite polyanalytic ensembles as WH ensembles with Hermite windows. This identification is carried out in Sect. 4 by means of symmetry arguments. The approximate identification of finite polyanalytic ensembles with finite WH ensembles is finished in Sect. 5 and gives a comparison of the processes defined by truncating the complex Hermite expansion on the one hand, and by the abstract concentration and spectral truncation method on the other. We explain the deviation between the two ensembles as stemming from two different quantization rules. The proof resorts to a Sobolev embedding for certain symbol classes known as modulation spaces. Some of the technical details are postponed to the appendix. Theorem 1.2 is proved in Sect. 5. In Sect. 6 we apply the symmetry argument from Sect. 4 to rederive the so-called simultaneous observability of polyanalytic ensembles. We also clarify the relation between the spectral expansions of the restriction and Toeplitz kernels. Finally, the appendix provides some background material on determinantal point processes, a certain symbol class for pseudo-differential operators, functions of bounded variation, and polyanalytic spaces.

## Harmonic Analysis on Phase Space

In this section we briefly discuss our tools. These methods from harmonic analysis are new in the study of determinantal point processes.

### The Short-Time Fourier Transform

Given a window function $g\in L^{2}({\mathbb {R}^{d}})$, the short-time Fourier transform of $f \in L^2({\mathbb {R}^d})$ is

2.1 $$\begin{aligned} V_{g}f(x,\xi )=\int _{{\mathbb {R}^d}} f(t) \overline{g(t-x)} e^{-2\pi i \xi t} dt, \qquad (x,\xi ) \in {\mathbb {R}^{2d}}. \end{aligned}$$

The short-time Fourier transform is closely related to the Schrödinger representation of the Heisenberg group, which is implemented by the operators

$$\begin{aligned} T(x,\xi ,\tau )g(t)=e^{2\pi i\tau }e^{-\pi ix\xi }e^{2\pi i\xi t}g(t-x), \qquad (x, \xi ) \in {\mathbb {R}^d}, \tau \in {\mathbb {R}}. \end{aligned}$$

The corresponding representation coefficients are

$$\begin{aligned} \left\langle f,T(x,\xi ,\tau )g\right\rangle =e^{-2\pi i\tau }e^{\pi ix\xi }\left\langle f,e^{2\pi i\xi \cdot }g(\cdot -x)\right\rangle = e^{-2\pi i\tau } e^{\pi ix\xi }V_g f(x,\xi ). \end{aligned}$$

As the variable $\tau$ occuring in the Schrödinger representation is unnecessary for DPPs, we will only use the short-time Fourier transform. We identify a pair $(x,\xi )\in {\mathbb {R}^{2d}}$ with the complex vector $z=x+i\xi \in {\mathbb {C}^{d} }$. In terms of the phase-space shifts in (1.12), the short-time Fourier transform is $V_{g}f(z):=\left\langle f,\pi (z)g\right\rangle$. The phase-space shifts satisfy the commutation relations

2.2 $$\begin{aligned} \pi (x,\xi )\pi (x^{\prime },\xi ^{\prime })=e^{-2\pi i\xi ^{\prime }x}\pi (x+x^{\prime },\xi +\xi ^{\prime }),\qquad (x,\xi ),(x^{\prime },\xi ^{\prime })\in {\mathbb {R}^{d}}\times {\mathbb {R}^{d}}, \end{aligned}$$

and the short-time Fourier transform satisfies the following * orthogonality relations* [32, Proposition 1.42] and [38, Theorem 3.2.1],

2.3 $$\begin{aligned} \left\langle V_{g_{1}}f_{1},V_{g_{2}}f_{2}\right\rangle _{L^2({{\mathbb {R}}^{2d}} )} =\left\langle f_{1},f_{2}\right\rangle _{L^2({\mathbb {R}}^d )} \overline{\left\langle g_{1},g_{2}\right\rangle }_{L^2({\mathbb {R}}^d )}. \end{aligned}$$

In particular, when $||g||_{2}=1$, the map $V_{g}$ is an isometry between $L^{2}({\mathbb {R}^{d}})$ and a closed subspace of $L^{2}({\mathbb {R} ^{2d}})$:

2.4 $$\begin{aligned} ||V_{g}f||_{{L^{2}({\mathbb {R}^{2d}})}}=||f||_{{L^{2}({ \mathbb {R}^{d}})}},\qquad f\in {L^{2}({\mathbb {R}^{d}})}. \end{aligned}$$

The commutation rule (2.2) implies the following formula for the short-time Fourier transform:

$$\begin{aligned} V_{g}(\pi (x,\xi )f)(x^{\prime },\xi ^{\prime })=e^{-2\pi ix(\xi ^{\prime }-\xi )}V_{g}f(x^{\prime }-x,\xi ^{\prime }-\xi ),\qquad (x,\xi ),(x^{\prime },\xi ^{\prime })\in {\mathbb {R}^{d}}\times {\mathbb {R}^{d}}. \end{aligned}$$

Since the phase-space shift of *f* on $\mathbb {R}^d$ corresponds to a phase-space shift of $V_gf$ on $\mathbb {R}^{2d}$, this formula is usually called the *covariance property* of the short-time Fourier transform.

### Special Windows

If we choose the Gaussian function $h_{0}(t)=2^{\frac{1}{4}}e^{-\pi t^{2}}$, $t \in {\mathbb {R}}$, as a window in (2.1), then a simple calculation shows that

2.5 $$\begin{aligned} e^{-i \pi x\xi + \frac{\pi }{2} \left| z\right| ^{2}}V_{h_{0}}f(x,-\xi )=2^{1/4} \int _{\mathbb {R}}f(t)e^{2\pi tz-\pi t^{2}- \frac{\pi }{2}z^{2}}dt=\mathcal {B}f(z), \end{aligned}$$

where $\mathcal {B}f(z)$ is the *Bargmann transform* of *f* [14, 32, Chapter 1.6]. The Bargmann transform $\mathcal {B }$ is a unitary isomorphism from $L^{2}({\mathbb {R}})$ onto the Bargmann-Fock space $\mathcal {F}({\mathbb {C}})$ consisting of all entire functions satisfying

2.6 $$\begin{aligned} \left\| F\right\| _{\mathcal {F}(\mathbb {C})}^{2}= \int _{\mathbb {C} }\left| F(z)\right| ^{2}e^{-\pi \left| z\right| ^{2}}dz<\infty . \end{aligned}$$

We now explain the relation between polyanalytic Fock spaces and phase-space analysis with Hermite windows $\{h_r: r\ge 0\}$. The *r*—*pure polyanalytic Bargmann transform* [2] is the map $\mathcal {B}^{r}:L^{2}({\mathbb {R}})\rightarrow L^{2}({\mathbb {C}},e^{-\pi \left| z\right| ^{2}})$

2.7 $$\begin{aligned} \mathcal {B}^{r}f(z):=e^{-i\pi x\xi +\tfrac{\pi }{2}\left| z\right| ^{2}}V_{h_{r}}f(x,-\xi ),\qquad z=x+i\xi . \end{aligned}$$

This map defines an isometric isomorphism between $L^{2}({\mathbb {R}})$ and the pure polyanalytic-Fock space $\mathcal {F}^{r}(\mathbb {C})$ (see Sect. A.5). The orthogonality relations (2.3) show that for $r\not =r^{\prime }$, $V_{h_{r}}f_{1}$ is orthogonal to $V_{h_{r^{\prime }}}f_{2}$ for all $f_1,f_2 \in L^{2}({\mathbb {R}})$. The relation between phase-space analysis and polyanalytic functions discovered in [2] can be understood in terms of the *Laguerre connection* [32, Chapter 1.9]:

2.8 $$\begin{aligned} V_{h_{r}} h_j (x,-\xi ) = e^{i\pi x\xi -\tfrac{\pi }{2}\left| z \right| ^2} H_{j,r}(z,{\bar{z}}), \end{aligned}$$

which, in terms of the polyanalytic Bargmann transform reads as

2.9 $$\begin{aligned} \mathcal {B}^{r}h_{j}(z)=H_{j,r}(z,{\bar{z}}), \end{aligned}$$

see also [2].

### The Range of the Short-Time Fourier Transform

For $||g||_{2}=1$, the short-time Fourier transform $V_{g}$ defines an isometric map $V_{g}:{L^{2}({\mathbb {R}^{d}})}\rightarrow {L^{2}({ \mathbb {R}^{2d}})}$ with range

$$\begin{aligned} {\mathcal {V}_{g}}:=\big \{\,V_{g}f\,:\,f\in {L^{2}({\mathbb {R}^{d}})}\,\big \} \subseteq {L^{2}({\mathbb {R}^{2d}})}. \end{aligned}$$

The adjoint of $V_{g}$ can be written formally as $V_{g}^{*}:L^{2}({ \mathbb {R}^{2d}})\rightarrow L^{2}({\mathbb {R}^{d}})$,

$$\begin{aligned} V_{g}^{*}F= \int _{{{\mathbb {R}}^{2d}} } F(z) \pi (z)g \, dz ,\qquad t\in {\mathbb {R}^{d}} \, , \end{aligned}$$

where the integral is to be taken as a vector-valued integral. The orthogonal projection $P_{\mathcal {V}_{g}}:L^{2}({\mathbb {R}^{2d}} )\rightarrow {\mathcal {V}_{g}}$ is then $P_{\mathcal {V}_{g}}=V_{g}V_{g}^{ *}$. Explicitly, $P_{\mathcal {V}_{g}}$ is the integral operator

$$\begin{aligned} P_{\mathcal {V}_{g}}F(z)=\int _{\mathbb {R}^{2d}} {K^g}(z,z^{\prime }) F(z^{\prime }) dz^{\prime },\qquad z=(x,\xi )\in {\mathbb {R}^{2d}}, \end{aligned}$$

where the *reproducing kernel*${K^g}$ is given by (1.1). Every function $F\in {\mathcal {V}_{g}}$ is continuous and satisfies the reproducing formula $F(z)=\int _{{{\mathbb {R}}^{2d}} } F(z^{\prime }){K^g} (z,z^{\prime })dz^{\prime }$.

### Metaplectic Rotation

We will make use of a rotational symmetry argument in phase space. Let $$ R_{\theta }:= \big [ {\begin{matrix} \cos (\theta ) &{} -\sin (\theta ) \\ \sin (\theta ) &{} \cos (\theta ) \end{matrix}} \big ]$$ denote the rotation by the angle $\theta \in {\mathbb {R}}$. The * metaplectic rotation* is the operator given in the Hermite basis $\left\{ h_{r}:r\ge 0\right\}$ by

2.10 $$\begin{aligned} {\mu (R_\theta )}f=\sum _{r\ge 0}e^{i r\theta }\left\langle f,h_{r}\right\rangle h_{r},\qquad f\in L^{2}(\mathbb {R}) \, , \end{aligned}$$

in particular, $\mu (R_\theta ) h_r = e^{i r\theta } h_r$. The standard and metaplectic rotations are related by

2.11 $$\begin{aligned} V_{g}f(R_{\theta }(x,\xi ))=e^{\pi i(x\xi -x{^{\prime }}\xi {^{\prime }} )}V_{\mu (R_{-\theta }) g}\mu (R_{-\theta }) f(x,\xi ), \text{ where } (x{^{\prime }},\xi {^{\prime }})=R_{\theta }(x,\xi ). \end{aligned}$$

This formula is a special case of the symplectic covariance of the Schrödinger representation; see [32, Chapters 1 and 2], [38, Chapter 9], or [23, Chapter 15]) for background and proofs.

### Time-Frequency Localization and Toeplitz Operators

Let us consider *g* with $||g||_{2}=1$. For $m\in L^{\infty }({\mathbb {R}^{2d}})$, the *Toeplitz operator*$M_{m}^{g}:{ \mathcal {V}_{g}}\rightarrow {\mathcal {V}_{g}}$ is

$$\begin{aligned} M_{m}^{g}F:=P_{\mathcal {V}_{g}}(m\cdot F),\qquad F\in {\mathcal {V}_{g}}, \end{aligned}$$

and its integral kernel at a point $(z,z')$ is given by

2.12 $$\begin{aligned} K_m(z,z^\prime )= \int _{{\mathbb {R}^{2d}}} {K^g} (z,z^{\prime \prime }) m (z^{\prime \prime }) {K^g} (z^{\prime \prime },z^{\prime }) \, dz^{\prime \prime }. \end{aligned}$$

When $m=1_\Omega$, the last expression coincides with (1.17). (The operator $M_{m}^{g}$ is defined on $\mathcal {V}_g$; the kernel in (2.12) represents the extension of $M_{m}^{g}$ to $L^2({\mathbb {R}^{2d}})$ that is 0 on $\mathcal {V}_g^\perp$.) Clearly, $||M_{m}^{g}||_{{\mathcal {V}_{g}}\rightarrow { \mathcal {V}_{g}}}\le ||m||_{\infty }$. In addition, it is easy to see that if $m\ge 0$, then $M_{m}^{g}$ is a positive operator. If $m\in L^1({{\mathbb {R}}^{2d}})$, then $M_m^g$ is trace-class. By (2.12) the trace of $M_m^g$ is

2.13 $$\begin{aligned} \mathrm {trace} (M_m^g ) = \int _{{\mathbb {R}}^{2d}}K_m(z,z) \, dz = \int _{{{\mathbb {R}}^{2d}}} \int _{{{\mathbb {R}}^{2d}}} |K^g(z,z^{\prime \prime })|^2 m(z^{\prime \prime }) \, dz dz^{\prime \prime } = \int _{{{\mathbb {R}}^{2d}}} m(z^{\prime \prime }) \, dz^{\prime \prime } \, , \end{aligned}$$

because the isometry property (2.4) implies that

$$\begin{aligned} \int _{{{\mathbb {R}}^{2d}}} |K^g(z,z^{\prime \prime })|^2 \, dz = \int _{{{\mathbb {R}}^{2d}}} |\langle \pi (z^{\prime \prime })g, \pi (z) g\rangle |^2 \, dz = 1 \, . \end{aligned}$$

The *time-frequency localization operator* with window *g* and symbol *m* is $H_{m}^{g}:=V_{g}^{*}M_{m}^{g}V_{g}:{L^{2}({\mathbb {R}^{d}})}\rightarrow { L^{2}({\mathbb {R}^{d}})}$. Hence $M_{m}^{g}$ and $H_{m}^{g}$ are unitarily equivalent.^5^ The situation is depicted in the following diagram.2.14Explicitly, the time-frequency localization operator applies a mask to the short-time Fourier transform:

$$\begin{aligned} H_{m}^{g}f:=\int _{{\mathbb {R}^{2d}}}m(z)V_{g}f(z)\pi (z)g\,dz, \qquad f \in L^2({{\mathbb {R}}^{2d}}). \end{aligned}$$

As we will use the connection between time-frequency localization on ${\mathbb {R}}^d$ and Toeplitz operators on ${{\mathbb {R}}^{2d}}$ in a crucial argument, we write   (2.14) as a formula

2.15 $$\begin{aligned} \langle H_m^g f, u\rangle&= \langle V_g (V_g^* M_m^g V_gf), V_gu\rangle \nonumber \\&= \langle P_{\mathcal {V}_g} (m \, V_gf), V_gu\rangle \nonumber \\&= \langle m \, V_gf ,V_gu \rangle \, . \end{aligned}$$

This formula makes sense for $f,u\in L^2({\mathbb {R}}^d)$ and $m\in L^\infty ({{\mathbb {R}}^{2d} } )$, but also for many other assumptions [21].

Time-frequency localization operators are useful in signal processing because they model time-varying filters. For Gaussian windows, they have been studied in signal processing by Daubechies [22] and as Toeplitz operators on spaces of analytic functions by Seip [56]; see also [6, Section 1.4]. When $m\in L^{1}({\mathbb {R}^{2d}})$, $H_{m}^{g}$ is trace-class by (2.13) and

2.16 $$\begin{aligned} \mathrm {trace}(H_{m}^{g})=\int _{{{\mathbb {R}}^{2d}} } m(z) dz \, . \end{aligned}$$

For more details see [21, 42, 43]. When $m=1_{\Omega }$, the indicator function of a set $\Omega$, we write $M_{\Omega }^{g}$ and $H_{\Omega }^{g}$. In this case, the positivity property implies that $0\le M_{\Omega }^{g}\le I$.

### The Weyl Correspondence

The *Weyl transform* of a distribution $\sigma \in \mathcal {S}^{\prime }({\mathbb {R}^{d}}\times {\mathbb {R}^{d}})$ is an operator $\sigma ^{w}$ that is formally defined on functions $f:{\mathbb {R}^{d}}\rightarrow { \mathbb {C}}$ as

$$\begin{aligned} \sigma ^{w} f(x):=\int _{{\mathbb {R}^{d}}\times {\mathbb {R}^{d}}}\sigma \left( \frac{x+y}{2},\xi \right) e^{2\pi i(x-y)\xi }f(y)dyd\xi ,\qquad x\in { \mathbb {R}^{d}}. \end{aligned}$$

Every continuous linear operator $T:\mathcal {S}({\mathbb {R}^{d}})\rightarrow \mathcal {S}^{\prime }({\mathbb {R}^{d}})$ can be represented in a unique way as $T=\sigma ^{w}$, and $\sigma$ is called its *Weyl symbol* (see [32, Chapter 2]). The Wigner distribution of a test function $g\in \mathcal {S}({\mathbb {R}^{d}})$ and a distribution $f\in \mathcal {S}^{\prime }({\mathbb {R}^{d}})$ is

$$\begin{aligned} W(f,g)(x,\xi )=\int _{{\mathbb {R}^{2d}}}f(x+\tfrac{t}{2})\overline{g(x-\tfrac{ t}{2})}e^{-2\pi it\xi }dt. \end{aligned}$$

The integral has to be understood distributionally. The map $(f,g)\mapsto W(f,g)$ extends to other function classes, for example, for $f,g\in L^{2}({ \mathbb {R}^{d}})$, *W*(*f*, *g*) is well-defined and

2.17 $$\begin{aligned} \left\| W(f,g)\right\| _2 = \left\| f\right\| _2 \left\| g\right\| _2. \end{aligned}$$

The Wigner distribution is closely related to the short-time Fourier transform:

$$\begin{aligned} W(f,g)(x,\xi )=2^{d}e^{4\pi ix\cdot \xi }V_{\tilde{g}}f(2x,2\xi ), \end{aligned}$$

where $\tilde{g}(x)=g(-x)$. The action of $\sigma ^{w}$ on a distribution can be easily described in terms of the Wigner distribution:

$$\begin{aligned} \left\langle \sigma ^{w}f,g\right\rangle =\left\langle \sigma ,W(g,f)\right\rangle . \end{aligned}$$

Time-frequency localization operators have the following simple description in terms of the Weyl calculus:

2.18 $$\begin{aligned} H_{m}^{g}=\left( m*W(g,g) \right) ^{w}. \end{aligned}$$

## Finite Weyl–Heisenberg Ensembles

### Definitions

To define finite Weyl–Heisenberg processes, we consider a domain $\Omega \subseteq {\mathbb {R}^{2d}}$ with non-empty interior, finite measure and finite perimeter, i.e., the characteristic function of $\Omega$ has bounded variation (see Sect. A.1). Since $M_{\Omega }^{g}$ is trace-class, the Toeplitz operator $M_{\Omega }^{g}$ can be diagonalized as

3.1 $$\begin{aligned} M_{\Omega }^{g}=\sum _{j\ge 1}\lambda _{j}^{\Omega }\, p_{g,j}^{\Omega } \otimes p_{g,j}^{\Omega },\qquad f\in {L^{2}({\mathbb {R}^{2d}})}, \end{aligned}$$

where $\left\{ {\lambda _{j}^{\Omega }}:j\ge 1\right\}$ are the non-zero eigenvalues of $M_{\Omega }^{g}$ in decreasing order and the corresponding eigenfunctions $\left\{ p_{g,j}^{\Omega }:j\ge 1\right\}$ are normalized in $L^{2}$. The operator $M_{\Omega }^{g}$ may have a non-trivial kernel, but it is known that it always has infinite rank [28, Lemma 5.8], therefore, the sequences $\{ {\lambda _{j}^{\Omega }}:j\ge 1\}$ and $\{p_{g,j}^{\Omega }: j \ge 1\}$ are indeed infinite. In addition, as follows from (2.16), we have

3.2 $$\begin{aligned} 0\le {\lambda _{j}^{\Omega }}\le 1, \text{ and } \sum _{j \ge 1}{ \lambda _{j}^{\Omega }}=\left| \Omega \right| . \end{aligned}$$

We remark that the eigenvalues $\lambda _{j}^{\Omega }$ do depend on the window function *g*. When we need to stress this dependence we write $\lambda _{j}(\Omega ,g)$.

*The finite Weyl–Heisenberg ensemble*$\mathcal {X}^g_\Omega$ is given by Definition 1.3. For technical reasons, we will also consider a more general class of WH ensembles depending on an extra ingredient. Given a subset $I\subseteq {\mathbb {N}}$, we let $\mathcal {X} ^g_{\Omega ,I}$ be the determinantal point process with correlation kernel

$$\begin{aligned} K_{g,\Omega ,I}(z,z^{\prime })=\sum _{j\in I}p_{g,j}^{\Omega }(z)\overline{ p_{g,j}^{\Omega }(z^{\prime })}. \end{aligned}$$

When $I=\{1,\ldots ,N_{\Omega }\}$ we obtain the finite WH ensemble $\mathcal {X}^g_\Omega$, while for $I={\mathbb {N}}$ we obtain the infinite ensemble. (In the latter case, the resulting point-process is independent of domain $\Omega$.) Later we need to analyze the properties of the ensemble $\mathcal {X}^g_{\Omega ,I}$ with respect to variations of the index set *I*. When no subset *I* is specified, we always refer to the ensemble $\mathcal {X} ^g_\Omega$ associated with $I=\{1,\ldots ,N_{\Omega }\}$.

#### Remark 3.1

The process $\mathcal {X}^g_{\Omega ,I}$ is well-defined due to the Macchi–Soshnikov theorem (see Sect. 1). Indeed, since the kernel $K_{g,\Omega ,I}$ represents an orthogonal projection, we only need to verify that it is locally trace-class. This follows easily from the facts that $0 \le K_{g,\Omega ,I}(z,z) \le {K^g}(z,z)=1$ and that the restriction operators are positive (see Sect. 6.1).

### Universality and Rates of Convergence

The one-point intensity associated with a Weyl–Heisenberg ensemble $\mathcal { X}^g_{\Omega ,I}$ is

$$\begin{aligned} \rho _{g,\Omega ,I}(z):=\sum _{j \in I}\left| p_{g,j}^{\Omega }(z)\right| ^{2}. \end{aligned}$$

For $\mathcal {X}^g_\Omega$, the intensity $\rho _{g,\Omega }$ has been studied in the realm of signal analysis, where it is known as the *accumulated spectrogram* [6, 7]. (Another interesting connection between DPP’s and signal analysis is the completeness results of Ghosh [35].) The results in [6, 7] imply Theorems 1.4 and 1.5, which apply to the finite Weyl–Heisenberg ensembles $\mathcal {X}^g_\Omega$. For the general ensemble $\mathcal {X}^g_{\Omega ,I}$ we have the following lemma.

#### Lemma 3.2

Let $\rho _{g,\Omega ,I}$ be the one-point intensity of a WH ensemble $\mathcal {X}^g_{\Omega ,I}$ with $\#I < \infty$. Then

$$\begin{aligned} ||\rho _{g,\Omega ,I}-1_\Omega ||_{L^1({\mathbb {R}^{2d}})} = \#I-\left| \Omega \right| + 2 \sum _{j \notin I} \lambda _{j}^{\Omega }. \end{aligned}$$

#### Proof

Using that $0\le \rho _{g,\Omega ,I} \le 1$ and (1.20) and (3.2), we first calculate

$$\begin{aligned} ||\rho _{g,\Omega ,I}-1_\Omega ||_{L^1(\Omega )}=\int _\Omega \left( 1-\rho _{g,\Omega ,I}(z)\right) \, dz = {|\Omega |} - \sum _{j \in I} \lambda _{j}^{\Omega }= \sum _{j \notin I} \lambda _{j}^{\Omega }. \end{aligned}$$

Second, since the eigenfunctions are normalized and $\int _\Omega |p_{g,j}^\Omega (z)|^2 \, dz = \lambda _j$, we have

$$\begin{aligned} ||\rho _{g,\Omega ,I}-1_\Omega ||_{L^1({\mathbb {R}^{2d}} \setminus \Omega )}&=\int _{{\mathbb {R}^{2d}}\setminus \Omega } \rho _{g,\Omega ,I}(z) \, dz = \sum _{j\in I} \int _{\mathbb {R}^{2d}} |p_{g,j}^\Omega (z)|^2 \, dz - \int _\Omega |p_{g,j}^\Omega (z)|^2 \, dz \\&= \sum _{j \in I} \left( 1-\lambda _{j}^{\Omega }\right) = \#I - \sum _{j \in I} \lambda _{j}^{\Omega }=\#I - \left| \Omega \right| + \sum _{j \notin I} \lambda _{j}^{\Omega }. \end{aligned}$$

The conclusion follows by adding both estimates. $\square$

## Hermite Windows and Polyanalytic Ensembles

### Eigenfunctions of Toeplitz Operators

We first investigate the eigenfunctions of Toeplitz operators with Hermite windows $\{h_r: r \ge 0\}$ and circular domains.

#### Proposition 4.1

Let $D_R \subseteq {\mathbb {R}}^2$ be a disk centered at the origin. Then the family of Hermite functions is a complete set of eigenfunctions for $H^{h_r}_{D_R}$. As a consequence, the set $\{H_{j,r}(z, \overline{z}) e^{-\pi |z|^2/2}: j \ge 0\}$ forms a complete set of eigenfunctions for ${\widetilde{M}}_{D_R}^{h_r}$ (where ${\widetilde{M}}_{D_R}^{h_r}$ is related to $M_{D_R}^{h_r}$ by (1.29).

#### Proof

Consider the metaplectic rotation $R_{\theta }$ with angle $\theta \in { \mathbb {R}}$ defined in (2.10). For $f,u\in L^{2}({\mathbb {R}})$, we use first (2.15) and then the covariance property in (2.11) and the rotational invariance of $D{_{R}}$ to compute:

$$\begin{aligned} \left\langle {\mu (R_\theta )}^{*}H_{D_R}^{h_r} {\mu (R_\theta )} f,u\right\rangle&=\left\langle H_{D_R}^{h_r}{\ \mu (R_\theta )}f,{ \mu (R_\theta )}u\right\rangle =\left\langle 1_{{D_{R}}}V_{h_{r}}{\mu (R_\theta ) }f,V_{h_{r}}{\ \mu (R_\theta )}u\right\rangle \\&=\left\langle 1_{D{_{R}}}V_{{\mu (R_\theta )}h_{r}} \mu (R_\theta ) f,V_{{ \mu (R_\theta )}h_{r}}{\mu (R_\theta )}u\right\rangle \\&=\left\langle 1_{D{_{R}}}V_{h_{r}}f(R_{-\theta } \,\cdot ), V_{h_{r}}u(R_{-\theta }\,\cdot )\right\rangle \\&=\int _{D{_{R}}}V_{h_{r}}f(z)V_{h_{r}}u(z)dz =\left\langle H_{D_R}^{h_r}f,u\right\rangle . \end{aligned}$$

We conclude that ${\mu (R_\theta )}^{*}H_{D_R}^{h_r}{\mu (R_\theta )} =H_{D_R}^{h_r}$, for all $\theta \in {\mathbb {R}}$. Applying this identity to a Hermite function gives

$$\begin{aligned} {\mu (R_\theta )}^{*} H_{D_R}^{h_r} h_j&= {\mu (R_\theta )}^{*} H_{D_R}^{h_r}{\mu (R_\theta )} \left( e^{-ij \theta } h_j \right) \\&= e^{-ij \theta } {\mu (R_\theta )}^{*} H_{D_R}^{h_r}{\mu (R_\theta )} h_j = e^{-ij \theta } H_{D_R}^{h_r} h_j. \end{aligned}$$

Thus, $$H_{D_R}^{h_r} h_j$$ is an eigenfunction of $${\mu (R_\theta )}^{*}$$ with eigenvalue $$e^{-ij \theta }$$. For irrational $$\theta $$, the numbers $$ \{e^{-ij \theta }: j \ge 0\}$$ are all different, and, therefore, the eigenspaces of $${\mu (R_\theta )}^{*}$$ are one-dimensional. Hence, $$ H_{D_R}^{h_r} h_j$$ must be a multiple of $$h_j$$. Thus, we have shown that each Hermite function is an eigenfunction of $$H_{D_R}^{h_r}$$. Since the family of Hermite functions is complete, the conclusion follows. The statement about the complex Hermite polynomials follows from (2.8) and (2.14); the extra phase-factors and conjugation bars disappear due to the renormalization $$M_{D_R}^{h_r} \mapsto {\widetilde{M}}_{D_R}^{h_r}$$. $$\square $$

### Eigenvalues of Toeplitz Operators

As a second step to identify polyanalytic ensembles as WH ensembles, we inspect the eigenvalues of Toeplitz operators.

#### Lemma 4.2

Let $R>0$. Then the eigenvalue of $H_{D_{R}}^{h_{r}}$ corresponding to $h_{j}$ and the eigenvalue of ${\widetilde{M}}_{D_R}^{h_r}$ corresponding to $H_{j,r}(z,\overline{z}) e^{-\pi |z|^2/2}$ are

4.1 $$\begin{aligned} \mu _{j,R}^{r}:= \left<H_{D_{R}}^{h_{r}} h_{j},h_j\right> =\int _{D_{R}}\left| H_{r,j}(z,{\bar{z}})\right| ^{2}e^{-\pi \left| z\right| ^{2}}dz. \end{aligned}$$

In particular, $\mu _{j,R}^{r}\not =0$ for all $j,r\ge 0$ and $R>0$, and

4.2 $$\begin{aligned} H_{D_{R}}^{h_{r}}&= \sum _{j \ge 0} \mu ^r_{j,R} \, h_j \otimes h_j. \end{aligned}$$

#### Proof

(4.1) follows immediately from the definitions. According to (1.4), $H_{r,j}$ vanishes only on a set of measure zero, thus we conclude that $\mu _{j,R}^{r}\not =0$. The diagonalization follows from Proposition 4.1. $\square$

#### Remark 4.3

Figure 4 shows a plot of $\mu ^1_{0,R}$ (solid, blue) and $\mu ^1_{1,R}$ (dashed, red) as a function of *R*. Note that for a certain value of *R*, the eigenvalue $\mu ^1_{0,R}=\mu ^1_{1,R}$ is multiple.

### Identification as a WH Ensemble

We can now identify finite pure polyanalytic ensembles as WH ensembles.

#### Proposition 4.4

Let $J \subseteq {\mathbb {N}}_0$ and $R>0$, then there exist a set $I \subseteq {\mathbb {N}}$ with $\#I=\#J$ such that

4.3 $$\begin{aligned} \left\{ V_{h_r} h_{j}: j \in J \right\} =\left\{ p_{h_{r},j}^{D_{R}}:j\in I\right\} . \end{aligned}$$

#### Proof

By Proposition 4.1 every Hermite function $h_j$ is an eigenfunction of $H^{h_r}_{D_R}$. In addition, by Lemma 4.2, the corresponding eigenvalue $\mu ^r_{j,R}$ is non-zero. Hence $V_{h_r} h_j$ is one of the functions $p_{h_r,j^{\prime }}^{D_R }$ in the diagonalization (3.1). The set $I := \{j^{\prime }: j \in J\}$ satisfies (4.3). $\square$

As a consequence, we obtain the following.

#### Proposition 4.5

The pure polyanalytic Ginibre ensemble with kernel $$ K_{r,N}$$ in (1.8) can be identified with a finite WH ensemble in the following way. Let $$D_{R_{N}}\subset {\mathbb {C}}$$ be the disk with area *N*. Let $$I_{r,N} \subseteq {\mathbb {N}}$$ be a set such that

4.4 $$\begin{aligned} \left\{ V_{h_r} h_{0}, \ldots , V_{h_r} h_{N-1} \right\} =\left\{ p_{h_{r},j}^{D_{R_N}}:j\in I_{r,N} \right\} , \end{aligned}$$

and $\# I_{r,N}=N$, whose existence is granted by Proposition 4.4. Then ${\widetilde{K}}_{h_r, D_{R_{N}}, I_{r,N}}=K_{r,N}$, and the corresponding point processes coincide. In particular

4.5 $$\begin{aligned} \rho _{r,N}(z)=\rho _{h_{r},D_{R_{N}},I_{r,N}}(z), \qquad z \in {\mathbb {C}}. \end{aligned}$$

#### Proof

Since $\# I_{r,N}=N$, we can write

$$\begin{aligned} K_{h_r, D_{R_{N}}}(z,z^{\prime }) = \sum _{j \in I_{r,N}} p_{h_{r},j}^{D_{R_N}}(z) \overline{p_{h_{r},j}^{D_{R_N}}(z^{\prime })} = \sum _{j=0}^{N-1} V_{h_r} h_{j}(z) \overline{V_{h_r} h_{j}(z^{\prime })}. \end{aligned}$$

Using (1.30) and (2.8) we conclude that

$$\begin{aligned} {\widetilde{K}}_{h_r, D_{R_{N}}}(z,z^{\prime }) = \sum _{j=0}^{N-1} H_{j,r}(z, \overline{z}) e^{-\pi |z|^2/2} \overline{H_{j,r}(z^{\prime },\overline{ z^{\prime }})} e^{-\pi |z^{\prime 2}/2} = K_{r,N}(z,z^{\prime }), \end{aligned}$$

as desired. This implies that the point processes corresponding to $K_{h_r, D_{R_{N}}}$ and $K_{r,N}$ are related by transformation $z \mapsto \overline{ z}$. Since $H_{j,r}(z,\overline{z})=\overline{H_{j,r}(\overline{z},z)}$, the intensities of the pure (*r*, *N*)-polyanalytic ensemble are invariant under the map $z \mapsto \overline{z}$ and the conclusion follows. $\square$

While Proposition 4.5 identifies finite pure polyanalytic ensembles with WH ensembles in the generalized sense of Sect. 3 , this is just a technical step. Our final goal is to compare finite polyanalytic ensembles with finite WH ensembles in the sense of Definition 1.3, where the index set is $I_{r,N}=\{1, \ldots , N\}$. Before proceeding we note that for the Gaussian $h_0$ such comparison is in fact an exact identification.

#### Corollary 4.6

For $r=0$, the set $I_{0,N}$ from Proposition 4.5 is $I_{0,N}=\{0, \ldots , N-1\}$. Thus, the *N*-dimensional Ginibre ensemble has the same distribution as the finite WH ensemble $\mathcal {X}^{h_0}_{D_{R_N}}$, and

4.6 $$\begin{aligned} \rho _{0,N}(z)=\rho _{h_{0},D_{R_{N}}}(z), \qquad z \in {\mathbb {C}}. \end{aligned}$$

#### Proof

The claim amounts to saying that the eigenvalues $\mu ^0_{j,R}$ in (4.1) are decreasing for all $R>0$, so that the ordering of the eigenfunctions in (3.1) coincides with the indexation of the complex Hermite polynomials. The explicit formula in (4.1) in the case $r=0$ gives the sequence of *incomplete Gamma functions*:

$$\begin{aligned} \mu ^0_{j,R} = \frac{1}{j!} \int _0^{\pi R^2} t^j e^{- t} dt = 1- e^{-\pi R^2} \sum _{k=0}^{j} \frac{\pi ^k}{k!} R^{2k}, \end{aligned}$$

which is decreasing in *j* (see for example [1, Eq. 6.5.13]). $\square$

## Comparison Between Finite WH and Polyanalytic Ensembles

Having identified finite pure polyanalytic ensembles as WH ensembles associated with a certain subset of eigenfunctions *I*, we now investigate how much this choice deviates from the standard one $I=\{1, \ldots , N\}$. Thus, we compare finite pure polyanalytic ensembles to the finite WH ensembles of Definition 1.3.

### Change of Quantization

As a main technical step, we show that the change of the window of a time-frequency localization operator affects the distribution of the corresponding eigenvalues in a way that is controlled by the perimeter of the localization domain. When *g* is a Gaussian, the map $m\mapsto H_{m}^{g}$ is called *Berezin’s quantization* or *anti-Wick calculus* [32, Chapter 2] or [49]. The results in this section show that if Berezin’s quantization is considered with respect to more general windows and in ${{\mathbb {R}}^{2d}}$, the resulting calculus enjoys similar asymptotic spectral properties. We consider the function class

5.1 $$\begin{aligned} {M^{1}}({\mathbb {R}^{d}}):=\big \{\,f\in L^{2}({\mathbb {R}^{d}})\,:\,||f||_{{M^{1}}}:=||V_{\phi }f||_{L^{1}({\mathbb {R}^{2d}} )}<+\infty \,\big \}, \end{aligned}$$

where $$\phi (x)=2^{d/4}e^{-\pi \left| x\right| ^{2}}$$. The class $$ M^{1}$$ is one of the *modulation spaces* used in signal processing. It is also important as a symbol-class for pseudo-differential operators. Indeed, the following lemma, whose proof can be found in [37], gives a trace-class estimate in terms of the $$M^{1}$$-norm of the Weyl symbol (see also [21, 42, 43]).

#### Proposition 5.1

Let $\sigma \in {M^1}({\mathbb {R}^{2d}})$. Then $\sigma ^{w}$ is a trace-class operator and

$$\begin{aligned} ||\sigma ^{w}||_{S^{1}}\lesssim ||\sigma ||_{{M^1}}, \end{aligned}$$

where $\left\| \cdot \right\| _{S^{1}}$ denotes the trace-norm.

The next lemma will allow us to exploit cancellation properties in the $M^1$-norm. Its proof is postponed to Sect. A.3.

#### Lemma 5.2

(A Sobolev embedding for $M^1$) Let $f\in L^{1}({\mathbb {R}^{d}})$ be such that $\partial _{x_{k}}f\in {M^1}({\mathbb {R}^{d}})$, for $k=1,\ldots ,d$. Then $f\in {M^1}({\mathbb {R}^{d}})$ and $||f||_{{M^1}}\lesssim ||f||_{L^{1}}+\sum _{k=1}^{d}||\partial _{x_{i}}f||_{{M^1}}$.

We can now derive the main technical result. Its statement uses the space of $\mathrm {BV}({\mathbb {R}^{2d}})$ of (integrable) functions of bounded variation; see Sect. A.1 for some background.

#### Theorem 5.3

Let $$g_{1},g_{2}\in \mathcal {S}({\mathbb {R}^d})$$ with $$ ||g_i||_{2}=1$$ and $$m\in \mathrm {BV}({\mathbb {R}^{2d}})$$. Then

$$\begin{aligned} ||H_m^{g_1} - H_m^{g_2}||_{S^{1}}\le C_{g_{1},g_{2}} var (m), \end{aligned}$$

where $C_{g_{1},g_{2}}$ is a constant that only depends on $g_1$ and $g_2$. In particular, when $m=1_\Omega$ we obtain that

$$\begin{aligned} ||H_\Omega ^{g_1} - H_\Omega ^{g_2}||_{S^{1}}\le C_{g_{1},g_{2}} \left| \partial \Omega \right| _{2d-1}. \end{aligned}$$

#### Proof of Theorem 5.3

Let us assume first that *m* is smooth and compactly supported. We use the description of time-frequency localization operators as Weyl operators. By (2.18), $$H_{m}^{g_{i}}=(m*W(g_{i},g_i))^{w}$$. Now, let $$ h:=W(g_{1},g_{1})-W(g_{2},g_{2})$$. Then $$h\in \mathcal {S}$$—see, e.g., [32, Proposition1.92]—and $$\int h=||g_{1}||_{2}^{2}-||g_{2}||_{2}^{2}=0$$ by (2.17). Hence, by Proposition 5.1,

$$\begin{aligned} ||H_{m}^{g_{1}}-H_{m}^{g_{2}}||_{S^{1}}=||(m*h)^{w}||_{S^{1}}\lesssim ||m*h||_{M^1}, \end{aligned}$$

Therefore, it suffices to prove that $||m*h||_{M^1}\lesssim var (m)$. We apply Lemma 5.2 to this end. First note that $\partial _{x_{i}}(m*h)=\partial _{x_{i}}m*h$ and, consequently,

$$\begin{aligned} ||\partial _{x_{i}}(m*h)||_{M^1}\lesssim ||\partial _{x_{i}}m||_{L^{1}}||h||_{M^1}\lesssim var (m). \end{aligned}$$

Second, we exploit the fact that $\int h=0$ to get

$$\begin{aligned} (m*h)(z)&=\int _{\mathbb {R}^d}m(z^{\prime })h(z-z^{\prime })dz^{\prime }=\int _{{\mathbb {R}^d}}(m(z^{\prime })-m(z))h(z-z^{\prime })dz^{\prime } \\&=\int _{{\mathbb {R}^d}}\int _{0}^{1}\left\langle \nabla (m)(tz^{\prime }+(1-t)z),z^{\prime }-z\right\rangle dt\,h(z-z^{\prime })dz^{\prime }, \end{aligned}$$

and consequently

$$\begin{aligned} \int _{{\mathbb {R}^d}}\left| m*h(z)\right| dz&\le \int _{0}^{1}\int _{{\mathbb {R}^d}}\int _{{\mathbb {R}^d}}\left| \nabla (m)(tz^{\prime }+(1-t)z)\right| \left| z^{\prime }-z\right| \left| h(z-z^{\prime })\right| dz^{\prime }dzdt \\&=\int _{0}^{1}\int _{{\mathbb {R}^d}}\int _{{\mathbb {R}^d}}\left| \nabla (m)(tw+z)\right| \left| w\right| \left| h(-w)\right| dwdzdt \\&=||\nabla m||_{L^{1}}\int _{0}^{1}\int _{{\mathbb {R}^d}}\left| w\right| \left| h(w)\right| dwdt= ||\nabla m||_{L^{1}}\int _{{\mathbb {R}^d}}\left| w\right| \left| h(w)\right| dw. \end{aligned}$$

Since $h\in \mathcal {S}$ the last integral is finite. We conclude that $||m*h||_{L^{1}}\lesssim ||\nabla m||_{L^{1}}= var (m)$, providing the argument for smooth, compactly supported *m*. For general $m \in \mathrm {BV}({\mathbb {R}^d})$, there exists a sequence of smooth, compactly supported functions $\left\{ m_k: k \ge 0 \right\}$ such that $m_k \rightarrow m$ in $L^1$, and $var (m_k) \rightarrow var (m)$, as $k \rightarrow +\infty$ (see for example [30, Sec. 5.2.2, Theorem 2].) By Proposition 5.1, $H^{g_i}_{m_k} \rightarrow H^{g_i}_m$ in trace norm, and the conclusion follows by a continuity argument. $\square$

### Comparison of Correlation Kernels

We now state and prove the main result on the comparison between finite WH ensembles associated with different subsets of eigenfunctions.

#### Theorem 5.4

Consider the identification of the (*r*, *N*)-pure polyanalytic ensemble as a finite WH ensemble with parameters $(h_r, D_{R_N}, I_{r,N})$ given by Proposition 4.5. Let $K_{h_r, D_{R_N},I_{r,N}}$ be the corresponding correlation kernel, and let $K_{h_r, D_{R_N}}$ be the correlation kernel of the finite Weyl–Heisenberg ensemble associated with the Hermite window $h_r$ and the disk $D_{R_N}$. Then

5.2 $$\begin{aligned} \bigl ||K_{h_r, D_{R_N}} - K_{h_r, D_{R_N},I_{r,N}}\bigr ||_{S^1} \lesssim \left| \partial D_{R_N} \right| _{1} \asymp \sqrt{N}, \end{aligned}$$

where $\left\| \cdot \right\| _{S^1}$ denotes the trace-norm of the corresponding integral operators.

#### Proof

*Step 1: Comparison of different polyanalytic levels.* We consider two eigen-expansions of the Toeplitz operator $M^{h_r}_{D_{R_N}}$:

5.3 $$\begin{aligned} M^{h_r}_{D_{R_N}}&= \sum _{j \ge 1} \lambda _j(D_{R_N}, h_r) \, p^{D_{R_N}}_{h_r, j} \otimes p^{D_{R_N}}_{h_r, j}, \end{aligned}$$

5.4 $$\begin{aligned} M^{h_r}_{D_{R_N}}&=\sum _{j \ge 0} \mu ^r_{j, R_N} \, V_{h_r} h_j \otimes V_{h_r} h_j. \end{aligned}$$

Recall that, while the eigenvalues in (5.4) are ordered non-increasingly, the eigenvalues in (5.3) follow the indexation of Hermite functions. When $r=0$, according to Corollary 4.6, the two expansions coincide: the sequence $\mu ^0_{j,R_N}$ is decreasing, and

5.5 $$\begin{aligned} \lambda _{j+1}(D_{R_N}, h_0)=\mu ^0_{j,R_N}, \qquad j \ge 0. \end{aligned}$$

We now quantify the deviation between the two eigen-expansions for general *r*. To this end, we use the unitary equivalence between $M^{h_r}_{D_{R_N}}$ and the time-frequency localization operator $H^{h_r}_{D_{R_N}}$—cf. (2.14). By (4.2),

$$\begin{aligned} H_{D_{R_{N}}}^{h_{r}}=\sum _{j\ge 0}\mu _{j,R_N}^{r} \, h_j \otimes h_j. \end{aligned}$$

While the operators $$M^{h_r}_{D_{R_N}}$$ act on mutually orthogonal subspaces of $$L^2({\mathbb {R}^{2d}})$$ for different values of *r*, their counterparts $$ H_{D_{R_{N}}}^{h_{r}}$$ act on configuration space and so can readily be compared by means of Theorem 5.3. We obtain

5.6 $$\begin{aligned} \left\| \mu _{\cdot , R_N} ^{0}-\mu _{\cdot , R_N} ^{r}\right\| _{\ell ^{1}}=\left\| H_{D_{R_{N}}}^{h_{0}}-H_{D_{R_{N}}}^{h_{r}}\right\| _{S^{1}}\le C_{r} \left| \partial D_{R_{N}} \right| _{1} \asymp R_N \asymp \sqrt{N}. \end{aligned}$$

*Step 2. Estimates for the spectral truncations.* According to Proposition 4.5,

5.7 $$\begin{aligned} K_{h_{r},D_{R_{N}},I_{r,N}} = \sum _{j=0}^{N-1} V_{h_r} h_j \otimes V_{h_r} h_j. \end{aligned}$$

For clarity, in what follows we denote by $T_K$ the operator with integral kernel *K*. Let $L_j := 1$ for $1 \le j \le N$ and $L_j := 0$, for $j>N$. Using the expansion in (5.4) and (3.1), we estimate the trace-norm:

$$\begin{aligned}&\left\| T_{K_{h_r, D_{R_N}}} - M^{h_r}_{D_{R_N}}\right\| _{S^1} = \Bigg \Vert { \sum _{j \ge 1} \big (L_j - \lambda _j(D_{R_N}, h_r)\big ) \, p^{D_{R_N}}_{h_r, j} \otimes p^{D_{R_N}}_{h_r, j} }\Bigg \Vert _{S^1} \\&\qquad \le \sum _{j \ge 1} \left| L_j - \lambda _j(D_{R_N}, h_r) \right| = \sum _{j=1}^N \left[ 1-\lambda _j(D_{R_N}, h_r) \right] + \sum _{j>N} \lambda _j(D_{R_N}, h_r) \\&\qquad = N - \sum _{j \ge 1} \lambda _j(D_{R_N}, h_r) + 2 \sum _{j>N} \lambda _j(D_{R_N}, h_r) = 2 \sum _{j>N} \lambda _j(D_{R_N}, h_r) \, , \end{aligned}$$

as $\sum _j \lambda _j = |D_{{R_N}}| = N$ by (3.2). Since $\phantom {\mu ^r_{j,R_{N_H}}}\mu ^r_{j,R_N}$ is a rearrangement of $\lambda _j(D_{R_N}, h_r)$, we can use (5.3) and (5.7) to mimic the argument. Thus, a similar calculation gives

$$\begin{aligned} \left\| T_{K_{h_r, D_{R_N}, I_{r,N}}} - M^{h_r}_{D_{R_N}}\right\| _{S^1} \le 2 \sum _{j>N-1} \mu ^r_{j,R_N}, \end{aligned}$$

and consequently,

5.8 $$\begin{aligned} \left\| T_{K_{h_r, D_{R_N}}} - T_{K_{h_r, D_{R_N}, I_{r,N}}}\right\| _{S^1} \lesssim \sum _{j>N} \lambda _j(D_{R_N}, h_r) + \sum _{j>N-1} \mu ^r_{j,R_N}. \end{aligned}$$

*Step 3. Final estimates* Combining (5.8) with (5.5) and (5.6) we obtain

5.9 $$\begin{aligned} \left\| T_{K_{h_r, D_{R_N}}} - T_{K_{h_r, D_{R_N}, I_{r,N}}}\right\| _{S^1} \lesssim \sum _{j>N} \lambda _j(D_{R_N}, h_r) + \sum _{j>N} \lambda _j(D_{R_N}, h_0) + \sqrt{N}. \end{aligned}$$

We now invoke Lemma 3.2 and Theorem 1.5 to estimate

5.10 $$\begin{aligned} \sum _{j>N} \lambda _j(D_{R_N}, h_r) \asymp \Big \Vert {\rho _{h_r, D_{R_N}} - 1_{D_{R_N}}\Big \Vert }_{L^1} \lesssim \left| \partial D_{R_{N}} \right| _{1} \asymp \sqrt{N}. \end{aligned}$$

Finally, (5.2) follows by combining (5.9) and (5.10). $\square$

### Transference to Finite Pure Polyanalytic Ensembles

#### Proof of Theorem 1.6

We use Proposition 4.5 to identify the (*r*, *N*)-polyanalytic ensemble with a Weyl–Heisenberg ensemble with parameters $$ (h_{r},D_{R_{N}},I_{r,N})$$, with correlation $$K_{h_{r},D_{R_{N}},I_{r,N}}$$ as in Theorem 5.4. By Proposition 4.5, $$ \widetilde{K}_{h_{r},D_{R_{N}},I_{r,N}} = K_{r,N}$$. Therefore, the conclusion follows from (5.2). $$\square $$

### The One-Point Intensity of Finite Polyanalytic Ensembles

#### Proof of Theorem 1.2

We use the notation of Theorem 5.4; in particular $R_N = \sqrt{ \tfrac{N}{\pi }}$. By (4.5), $\rho _{r,N}= \rho _{h_{r},D_{R_{N}},I_{r,N}}$, and we can estimate

$$\begin{aligned} \Big \Vert \rho _{r,N}-1_{D_{R_N}}\Big \Vert _{1} \le \Big \Vert \rho _{h_{r},D_{R_{N}},I_{r,N}} - \rho _{h_r,D_{R_N}}\Big \Vert _1 + \left\| \rho _{h_r,D_{R_N}} - 1_{D_{R_N}}\right\| _1. \end{aligned}$$

By Theorem 1.5, $\left\| \rho _{h_r,D_{R_N}} - 1_{D_{R_N}}\right\| _1 \lesssim \sqrt{N}$. In addition, by Lemma A.1 in the appendix,

$$\begin{aligned} \left\| \rho _{h_{r},D_{R_{N}},I_{r,N}} - \rho _{h_r,D_{R_N}}\right\| _1&= \int _{{\mathbb {R}^{2d}}} \left| K_{h_{r},D_{R_{N}},I_{r,N}}(z,z) - K_{h_r,D_{R_N}}(z,z) \right| dz \\&\le \bigl ||K_{h_{r},D_{R_{N}},I_{r,N}} - K_{h_r,D_{R_N}}\bigr ||_{S^1}. \end{aligned}$$

Hence, the conclusion follows from Theorem 5.4. $\square$

Note that the proofs of Theorems 5.4 and 1.2 combine our main insights: the identification of the finite polyanalytic ensembles with certain WH ensembles, the analysis of the spectrum of time-frequency localization operators and Toeplitz operators, and the non-asymptotic estimates of the accumulated spectrum.

## Double Orthogonality

### Restriction Versus Localization

Let $\mathcal {X}^g$ be an infinite WH ensemble on ${\mathbb {R}^{2d}}$ and $\Omega \subseteq {\mathbb {R}^{2d}}$ of finite measure and non-empty interior. We consider the *restriction operator*$T^g_\Omega : L^2({\mathbb {R}^{2d}}) \rightarrow L^2( {\mathbb {R}^{2d}})$,

$$\begin{aligned} T^g_\Omega F := 1_\Omega P_{\mathcal {V}_g} (1_\Omega \cdot F), \end{aligned}$$

and the *inflated Toeplitz operator*$S^g_\Omega : L^2({\mathbb {R}^{2d}}) \rightarrow L^2( {\mathbb {R}^{2d}})$,

$$\begin{aligned} S^g_\Omega F := P_{\mathcal {V}_g} (1_\Omega \cdot P_{\mathcal {V}_g} F). \end{aligned}$$

In view of the decomposition $L^2({\mathbb {R}^{2d}}) = \mathcal {V}_{g} \oplus \mathcal {V }_{g}^\perp$, $S^g_\Omega$ and $M_{\Omega }^{g}$ are related by

$$\begin{aligned} S^g_{\Omega } = \left[ \begin{array}{cc} M_{\Omega }^{g} &{}\quad 0 \\ 0 &{}\quad 0 \end{array} \right] , \end{aligned}$$

and therefore share the same non-zero eigenvalues, and the corresponding eigenspaces coincide. The integral representation of $S^g_\Omega$ is given by (1.17). Since $P_{\mathcal {V}_g}$ and $F \mapsto F \cdot 1_\Omega$ are orthogonal projections, both $T^g_\Omega$ and $S^g_\Omega$ are self-adjoint operators with spectrum contained in [0, 1]. The integral kernel of $T^g_\Omega$ is given by (1.14) and $\int {K^g} _{|\Omega }(z,z) dz = \left| \Omega \right| <+\infty$. Therefore, $T^g_\Omega$ is trace-class (see e.g. [58, Theorems 2.12 and 2.14]). It is an elementary fact that $T^g_\Omega$ and $S^g_\Omega$ have the same non-zero eigenvalues with the same multiplicities (this is true for *PQP* and *QPQ* whenever *P* and *Q* are orthogonal projections). Morever, for $\lambda \not =0$, the map

$$\begin{aligned} F \longmapsto \frac{1}{\sqrt{\lambda }} 1_\Omega F \end{aligned}$$

is an isometry between the eigenspaces

$$\begin{aligned} \left\{ F \in L^2({\mathbb {R}^{2d}}): S^g_\Omega F = \lambda F \right\} \longrightarrow \left\{ F \in L^2({\mathbb {R}^{2d}}): T^g_\Omega F = \lambda F \right\} \, . \end{aligned}$$

Therefore, if $M^g_\Omega$ is diagonalized as in (1.18), then $T^g_\Omega$ can be expanded as in (1.19). This justifies the discussion in Sect. 1.3.

### Simultaneous Observability

Let $\mathcal {X}$ be a determinantal point process (with a Hermitian locally trace-class correlation kernel). We say that a family of sets $\left\{ \Omega _\gamma :\gamma \in \Gamma \right\}$ is * simultaneously observable* for $\mathcal {X}$, if the following happens. Let $\Omega =\bigcup _{\gamma \in \Gamma }\Omega _\gamma$. There is an orthogonal basis $\{\varphi _{j}:j\in J\}$ of the closure of the range of the restriction operator $T_\Omega$ consisting of eigenfunctions of $T_{\Omega }$ such that for each $\gamma \in \Gamma$, the set $\{\varphi _{j}|_{\Omega _ \gamma }:j\in J\}$ of the restricted functions is orthogonal. This is a slightly relaxed version of the notion in [44, p. 69]: in the situation of the definition, the functions $\{\varphi _{j}|_{\Omega _\gamma }:j\in J\} \setminus \left\{ 0 \right\}$ form an orthogonal basis of the closure of the range of $T_{\Omega _\gamma }$, but we avoid making claims about the kernel of $T_\Omega$. As explained in [44, p. 69], the motivation for this terminology comes from quantum mechanics, where two physical quantities can be measured simultaneously if the corresponding operators commute (or, more concretely, if they have a basis of common eigenfunctions).

#### Theorem 6.1

Let $\mathcal {D} = \left\{ D_R: R \in {\mathbb {R}}^+ \right\}$ be the family of all disks of ${\mathbb {R}}^2$ centered at the origin and $r\in {\mathbb {N}}$. Then

(i) $\mathcal {D}$ is simultaneously observable for the infinite Weyl–Heisenberg ensemble with window $h_{r}$.
(ii) Let $D_{R_0}$ be a disk and $I\subseteq {\mathbb {N}}$. Then $\mathcal {D}$ is simultaneously observable for the Weyl–Heisenberg ensemble $\mathcal {X}^{h_r}_{D_{R_0}, I}$.

#### Proof

Let us prove (i). Since the definition of simultaneous observability involves the orthogonal complement of the kernels of the restriction operators $T^g_{D_R}$, $\overline{\mathrm {ran}} (T_{D_R}^g) = (\mathrm {ker} \, T_{D_R}^g)^\perp$, the discussion in Sect. 6.1 implies that it suffices to show that the Toeplitz operators $M_{D_R}^{h_r}$ have a common basis of eigenfunctions. Since $V_{h_r}^{*}M_{D_R}^{h_r}V_{h_r}=H_{D_R}^{h_r}$, and, by Proposition 4.1, the Hermite basis diagonalizes $H_{D_R}^{h_r}$ for all $R>0$, the conclusion follows.

Let us now prove (ii). The ensemble $\mathcal {X}^{h_r}_{D_{R_0}, I}$ is constructed by selecting the eigenfunctions of the Toeplitz operator $M_{D_{R_0}}^{h_r}:{\mathcal {V}_{h_r}}\rightarrow {\mathcal {V}_{h_r}}$ corresponding to the indices in *I*:

$$\begin{aligned} K^{h_r}_{D_{R_0},I}(z,z^{\prime })=\sum _{j\in I}p^{D_{R_0}}_{h_r,j}(z) \overline{p^{D_{R_0}}_{h_r,j}(z^{\prime })}. \end{aligned}$$

Since, by part (i), the functions $p^\Omega _{g,j}$ are orthogonal when restricted to disks, the conclusion follows. $\square$

As a consequence, we obtain Theorem 1.7, which we restate for convenience.

#### Theorem 1.7

The family $\mathcal {D} = \left\{ D_R: r \in {\mathbb {R}}^+ \right\}$ of all disks of ${\mathbb {C}}$ centered at the origin is simultaneously observable for every finite and infinite pure-type polyanalytic ensemble.

#### Proof

This follows immediately from Proposition 4.5 and Theorem 6.1. (This slightly extends a result originally derived by Shirai [57].) $\square$

### An Extension of Kostlan’s Theorem

Theorem 1.8 is a consequence of the following slightly more general result.

#### Theorem 6.2

Let $\mathcal {X}$ be the determinantal point process associated with the (*r*, *J*)-pure polyanalytic ensemble, with $J\subseteq {\mathbb {N}}_{0}$ finite. Then the point process on $[0,+\infty )$ of absolute values $\left| \mathcal {X}\right|$ has the same distribution as the process generated by $\{Y_{j}: j\in J\}$ where the $Y_{j}$’s are independent random variables with density

$$\begin{aligned} f_{Y_{j}}(x):=2 \frac{\pi ^{j-r+1} r!}{j!} x^{2(j-r)+1}\left[ L_{r}^{j-r}(\pi x^2)\right] ^{2}e^{-\pi x^2}. \end{aligned}$$

(Hence, $Y^2_j$ is distributed according to $f_{Y_j^2}(x) = \frac{\pi ^{j-r+1} r!}{j!} x^{j-r}\left[ L_{r}^{j-r}(\pi x)\right] ^{2}e^{-\pi x}$.)

#### Proof

We want to show that the point processes $$\left| \mathcal {X}\right| :=\sum _{x\in \mathcal {X}}\delta _{\left| x\right| }$$ on $$\mathbb {R}$$ and $$\mathcal {Y} :=\sum _{j\in J}\delta _{Y_{j}}$$ on $$\mathbb {C}$$ have the same distribution. Let $$ I_{k}=[r_{k},R_{k}]$$, $$k=1,\ldots N$$, be a disjoint family of subintervals of $$[0,+\infty )$$. Then

$$\begin{aligned} \left( \mathcal {Y}(I_{1}),\ldots ,\mathcal {Y}(I_{N})\right) \overset{d}{=} \sum _{j\in J}\zeta _{j}, \end{aligned}$$

where the $\zeta _{j}$ are independent, $\mathbb {P}(\zeta _{j}=e_{k})= \int _{r_{k}}^{R_{k}}f_{Y_{j}}(x)dx$, and $\mathbb {P}(\zeta _{j}=0)=\int _{{\mathbb {R}}\setminus \cup _{k}[r_{k},R_{k}]}f_{Y_{j}}(x)dx$. On the other hand, Theorem 1.7 implies that the annuli $A_k:= \left\{ z \in {\mathbb {C}}: r_k \le \left| z \right| \le R_k \right\}$ are simultaneously observable for $\mathcal {X}$. Hence, by [44, Proposition 4.5.9]—which is still applicable for the slightly more general definition of simultaneous observability in Sect. 6.2, we have

$$\begin{aligned} \left( \left| \mathcal {X}\right| (I_{1}),\ldots ,\left| \mathcal { \ X}\right| (I_{N})\right) =\left( \mathcal {X}(A_{1}),\ldots ,\mathcal {X} (A_{N})\right) \overset{d}{=}\sum _{j\in J}\zeta _{j}^{\prime }, \end{aligned}$$

where the $\zeta _{j}^{\prime }$ are independent, $\mathbb {P} (\zeta _{j}^{\prime }=e_{k})= \int _{A_k}\left| H_{j,r}(z,\overline{z} )\right| ^{2} e^{-\pi \left| z\right| ^{2}} dz$, and $\mathbb {P} (\zeta _{j}^{\prime }=0) = \int _{{\mathbb {C}}\setminus \cup _k A_k}\left| H_{j,r}(z,\overline{z})\right| ^{2} e^{-\pi \left| z\right| ^{2}} dz$. A direct calculation, together with the identity

$$\begin{aligned} \frac{(-x)^{k}}{k!}L_{r}^{k-r}(x)=\frac{(-x)^{r}}{r!}L_{k}^{r-k}(x) \end{aligned}$$

shows that $\left( \zeta _{j}: j \in J \right) \overset{d}{=} \left( \zeta _{j}^{\prime }: j \in J \right)$ and the conclusion follows. $\square$

#### Remark 6.3

Let *n*(*R*) denote the number of points of a point process in the disk of radius *R* centered at the origin. An immediate consequence of Theorem 6.2 is the following formula for the probability of finding such a disk void of points, when the points are distributed according to the a polyanalytic Ginibre ensemble of the pure type:

$$\begin{aligned} \mathbb {P}\left[ n(R)=0\right] =\prod _{j}P\left( Y_{j} \ge R \right) \end{aligned}$$

This is known as the hole probability (see [44, Section 7.2] for applications in the case of the Ginibre ensemble).

## Appendix A: Additional Background Material

### Determinantal Point Processes and Intensities

We follow the presentation of [15, 44]. Let $K: {\mathbb {R}}^d \times {\mathbb {R}}^d \rightarrow {\mathbb {C}}$ be a locally trace-class Hermitian kernel with spectrum contained in [0, 1], and consider the functions

A.1 $$\begin{aligned} \rho _{n}(x_1, \ldots , x_n) := \det \left( K(x_j, x_k) \right) _{j,k=1,\ldots ,d}, \qquad x_1, \ldots , x_n \in {\mathbb {R}^d}. \end{aligned}$$

The Macchi-Soshnikov theorem implies that there exists a point process $\mathcal {X}$ on ${\mathbb {R}}^d$ such that for every family of disjoint measurable sets $\Omega _1, \ldots \Omega _n \subseteq {\mathbb {R}}^d$,

$$\begin{aligned} \mathbb {E} \left[ \prod _{j=1}^n \mathcal {X}(\Omega _j) \right] = \int _{\prod _j \Omega _j} \rho _{n}(x_1,\ldots ,x_n) dx_{1} \ldots dx_n, \end{aligned}$$

where $\mathcal {X}(\Omega )$ denotes the number of points of $\mathcal {X}$ to be found in $\Omega$. The functions $\rho _{n}$ are known as correlation functions or intensities and $\mathcal {X}$ is called a determinantal point process. The one-point intensity $\rho$ is simply the diagonal of the correlation kernel

$$\begin{aligned} \rho (x) = \rho _{1}(x)=K(x,x), \end{aligned}$$

and allows one to compute the expected number of points to be found on a domain $\Omega$:

$$\begin{aligned} \mathbb {E}\left[ \mathcal {X}(\Omega )\right] =\int _{\Omega }\rho (x)dx. \end{aligned}$$

The one-point intensity can also be used to evaluate expectations of linear statistics:

$$\begin{aligned} \tfrac{1}{n}\mathbb {E} \left[ f(x_{1})+\cdots +f(x_{n})\right] =\mathbb {E}\left[ f(x_{1})\right] =\int _{{\mathbb {R}^d}}f(x)\rho (x)dx. \end{aligned}$$

A DPP can be represented by different kernels. If $m: {\mathbb {R}}^d \rightarrow {\mathbb {C}}$ is unimodular (i.e., $\left| m(z) \right| =1$), then the kernel

$$\begin{aligned} K_m(x,x') = \overline{m}(x) K(x,x') m(x'), \end{aligned}$$

produces the same intensities in (A.1) as *K* does. (This is a so-called *gauge transformation*). The integral operator with kernel $K_m$ is related to the one with kernel *K* by

$$\begin{aligned} \overline{m}(x) T_K (m f) (x) = \int _{{\mathbb {R}^d}} \overline{m}(x) K(x,x') m(x') f(x') dx' = T_{K_m} f(x). \end{aligned}$$

Similarly, a linear transformation of a DPP corresponds to a linear change of variables in the kernel *K*.

### Functions of Bounded Variation

A real-valued function $f\in L^{1}({\mathbb {R}^{d}})$ is said to have *bounded variation*, $f\in \mathrm {BV}({\mathbb {R}^{d}})$, if its distributional partial derivatives are finite Radon measures. The variation of *f* is defined as

$$\begin{aligned} var (f):=\sup \left\{ \int _{\mathbb {R}^{d}}f(x)\, \mathrm {div}\, \phi (x)dx:\phi \in C_{c}^{1}({\mathbb {R}^{d}},{\mathbb {R}^{d}}),\left| \phi (x)\right| _{2}\le 1\right\} , \end{aligned}$$

where $C_{c}^{1}({\mathbb {R}^{d}},{\mathbb {R}^{d}})$ denotes the class of compactly supported $C^{1}$-vector fields and $\mathrm {div}$ is the divergence operator. If *f* is continuously differentiable, then $f\in \mathrm {BV}({\mathbb {R}^{d}})$ simply means that $\partial _{x_{1}}f,\ldots$ , $\partial _{x_{d}}f\in L^{1}({\mathbb {R}^{d}})$, and $var (f)=\int _{ \mathbb {R}^{d}}\left| \nabla f(x)\right| _{2}dx=||\nabla f||_{L^{1}}$. A set $\Omega \subseteq {\mathbb {R}^{d}}$ is said to have *finite perimeter* if its characteristic function $1_{\Omega }$ is of bounded variation, and the perimeter of $\Omega$ is defined as $\left| \partial \Omega \right| _{d-1} := var (1_{\Omega })$. If $\Omega$ has a smooth boundary, then $\left| \partial \Omega \right| _{d-1}$ is just the $(d-1)$-Hausdorff measure of the topological boundary. See [30, Chapter 5] for an extensive discussion of $\mathrm {BV}$.

### Trace-Class Operators

#### Lemma A.1

Let $K: {\mathbb {R}^d}\times {\mathbb {R}^d}\rightarrow {\mathbb {C}}$ be a continuous function and assume that the integral operator

$$\begin{aligned} T_K f(x) = \int _{\mathbb {R}^d}K(x,y) f(y) dy, \qquad f \in L^2({\mathbb {R}^d}), \end{aligned}$$

is well-defined, bounded, and trace-class. Then $\int _{\mathbb {R}^d}\left| K(x,x) \right| dx \le \left\| T_K\right\| _{S^1}$, where $\left\| \cdot \right\| _{S^1}$ denotes the trace-norm.

#### Proof

Let $T_K = \sum _j \mu _j \varphi _j \otimes \psi _j$, with $\mu _j \ge 0$ and $\{\varphi _j: j \ge 1\}$, $\{\psi _j: j \ge 1\}$ orthonormal. Then $K(x,y) = \sum _j \mu _j \varphi _j(x) \overline{\psi _j(y)}$ for almost every (*x*, *y*), and we can formally compute

$$\begin{aligned} \int _{\mathbb {R}^d}\left| K(x,x) \right| dx&\le \sum _j \mu _j \int _{\mathbb {R}^d}\left| \varphi _j(x) \right| \left| \psi _j(x) \right| dx \\&\le \sum _j \mu _j \left( \int _{\mathbb {R}^d}\left| \varphi _j(x) \right| ^2 dx \right) ^{1/2} \left( \int _{\mathbb {R}^d}\left| \psi _j(x) \right| ^2 dx \right) ^{1/2} \\&=\sum _j \mu _j = \left\| T_K\right\| _{S^1}. \end{aligned}$$

An approximation argument using the continuity of *K* is needed to justify the computations with the restriction of *K* to the diagonal—see [58, Chapters 1,2,3] for related arguments. $\square$

### Properties of Modulation Spaces

Recall the definition of the modulation space $M^1$ in (5.1). It is well-known that, instead of the Gaussian function $\phi$, any non-zero Schwartz function can be used to define ${M^{1}}$, giving an equivalent norm [31, 38, Chapter 9]. Using this fact, the following lemma follows easily.

#### Lemma A.2

Let $$f\in {L^{2}({\mathbb {R}^{d}})}$$. Then:

(i) $f\in {M^1}({\mathbb {R}^{d}})$ if and only if ${\hat{f}}\in {M^1}({ \mathbb {R}^{d}})$, where ${\hat{f}}$ is the Fourier transform of *f*. In this case: $||f||_{{M^1}}\asymp ||{\hat{f}}||_{{M^1}}$.
(ii) If *f* is supported on $D_1(0)=\{x:\left| x \right| \le 1\}$ and ${\hat{f}}\in L^{1}({\mathbb {R}^{d}})$, then $f\in {M^1}({\mathbb {R}^{d}})$ and $||f||_{{M^1}}\lesssim ||{\hat{f}}||_{L^{1}}$.
(iii) If $f\in {M^1}({\mathbb {R}^{d}})$ and $m\in C^{\infty }({ \mathbb {R}^{d}})$ has bounded derivatives of all orders, then $m\cdot f\in { M^1}({\mathbb {R}^{d}})$, and $||m\cdot f||_{{M^1}}\le C_{m}||f||_{{M^1}}$, where $C_{m}$ is a constant that depends on *m*.

We now prove the Sobolev embedding lemma that was used in Sect. 5.1.

#### Proof of Lemma 5.2

Let *g* be such that ${\hat{g}}=f$. By Lemma A.2, it suffices to show that $g\in {M^{1}}(\mathbb {R})$ and satisfies a suitable norm estimate. Let $\eta \in C^{\infty }(\mathbb {R})$ be such that $\eta (\xi )\equiv 0$ for $\left| \xi \right| \le 1/2$ and $\eta (\xi )\equiv 1$ for $\left| \xi \right| >1$. We write $\eta (\xi )=\sum _{k=1}^{d}\xi _{k}\eta _{k}(\xi )$, where $\eta _{k}\in C^{\infty }(\mathbb {R})$ has bounded derivatives of all orders. We set $g_{1}:=\eta \cdot g$ and $g_{2}:=(1-\eta )\cdot g$. Then $g_{1}(\xi )=\sum _{k=1}^{d}\eta _{k}(\xi )\xi _{k}g(\xi )$. Since $\xi _{k}g(\xi )=\tfrac{1}{2\pi i}\widehat{\partial _{x_{k}}f}(\xi )$ is in $M^1$ by Lemma A.2(i) and $\eta _{k}$ has bounded derivatives of all orders, we conclude from Lemma A.2 (iii) that $g_{1}\in {M^{1}}(\mathbb {R})$ and that

$$\begin{aligned} \left\| g_1\right\| _{M^1} \asymp \left\| \widehat{g}_1\right\| _{M^1} \lesssim \sum _{k=1}^{d} \left\| \xi _k \widehat{g}\right\| _{M^1} \asymp \sum _{k=1}^{d}||\partial _{x_{k}}f||_{{M^{1}}}. \end{aligned}$$

On the other hand, since *g* has an integrable Fourier transform, so does $g_{2}=(1-\eta )\cdot g$ and $||\widehat{g_{2}}||_{L^{1}}\lesssim ||f||_{L^{1}}$. In addition, $g_{2}$ is supported on $D_{1}(0)$. Therefore, by Lemma A.2, $g_{2}\in {M^{1}}$ and $||g_{2}||_{{M^{1}}}\lesssim ||f||_{L^{1}}$. Hence $g=g_{1}+g_{2}\in {M^{1}}$, and it satisfies the stated estimate. $\square$

### Polyanalytic Bargmann-Fock Spaces

A complex-valued function $F(z,\overline{z})$ defined on a subset of $\mathbb {C}$ is said to be *polyanalytic of order *$q-1$, if it satisfies the generalized Cauchy–Riemann equations

A.2 $$\begin{aligned} \left( \partial _{\overline{z}}\right) ^{q}F(z,\overline{z})=\frac{1}{2^{q}} \left( \partial _{x}+i\partial _{\xi }\right) ^{q}F(x+i\xi ,x-i\xi )=0 \, . \end{aligned}$$

Equivalently, *F* is a polyanalytic function of order $q-1$ if it can be written as

A.3 $$\begin{aligned} F(z,\overline{z})=\sum _{k=0}^{q-1}\overline{z}^{k}\varphi _{k}(z), \end{aligned}$$

where the coefficients $\{\varphi _{k}(z)\}_{k=0}^{q-1}$ are analytic functions. The *polyanalytic Fock space*${\mathbf {F}}^{q}(\mathbb {C})$ consists of all the polyanalytic functions of order $q-1$ contained in the Hilbert space $L^{2}(\mathbb {C},e^{-\pi \left| z\right| ^{2}})$. The reproducing kernel of the polyanalytic Fock space ${\mathbf {F}}^{q}( \mathbb {C})$ is

$$\begin{aligned} {\mathbf {K}}^{q}(z,z^{\prime })=L_{q}^{1}(\pi \left| z-z^{\prime }\right| ^{2})e^{\pi z \overline{z^{\prime }}}. \end{aligned}$$

Polyanalytic Bargmann-Fock spaces appear naturally in vector-valued time-frequency analysis [2, 39] and signal multiplexing [12, 13]. Within ${\mathbf {F}}^{q}(\mathbb {C})$ we distinguish the * polynomial subspace*

$$\begin{aligned} Pol_{\pi ,q,N}=span\{z^{j}\overline{z}^{l}:0\le j\le N-1,0\le l\le q-1\}, \end{aligned}$$

with the Hilbert space structure of $L^{2}(\mathbb {C},e^{-\pi \left| z\right| ^{2}})$. The polyanalytic Ginibre ensemble, introduced in [40], is the DPP with correlation kernel corresponding to the orthogonal projection onto $Pol_{\pi ,q,N}$ (weighted with the Gaussian measure). In [40, Proposition 2.1] it is shown that

$$\begin{aligned} Pol_{\pi ,q,N}=span\{H_{j,r}(z,\overline{z}):0\le j\le N-1,0\le r\le q-1\}, \end{aligned}$$

where $H_{j,r}$ are the complex Hermite polynomials (1.4). Thus, the reproducing kernel of $Pol_{\pi ,q,N}$ can be written as

A.4 $$\begin{aligned} {\mathbf {K}}_{\pi ,N}^{q}(z,z^{\prime })=\sum _{r=0}^{q-1}\sum _{j=0}^{N-1}H_{j,r}(z,\overline{z}) \overline{ H_{j,r}(z^{\prime },\overline{z^{\prime }})}. \end{aligned}$$

### Pure Polyanalytic-Fock Spaces

The pure polyanalytic Fock spaces $\mathcal {F}^{r}(\mathbb {C})$ have been introduced by Vasilevski [63], under the name of true polyanalytic spaces. They are spanned by the complex Hermite polynomials of fixed order *r* and can be defined as the set of polyanalytic functions *F* integrable in $L^{2}(\mathbb {C},e^{-\pi \left| z\right| ^{2}})$ and such that, for some entire function *H* [2],

$$\begin{aligned} F(z)=\left( \frac{\pi ^{r}}{r!}\right) ^{\frac{1}{2}}e^{\pi \left| z\right| ^{2}}\left( \partial _{z}\right) ^{r}\left[ e^{-\pi \left| z\right| ^{2}}H(z)\right] . \end{aligned}$$

Vasilevski [63] obtained the following decomposition of the polyanalytic Fock space ${\mathbf {F}}^{q}(\mathbb {C})$ into pure components

A.5 $$\begin{aligned} {\mathbf {F}}^{q}(\mathbb {C})=\mathcal {F}^{0}(\mathbb {C})\oplus \cdots \oplus \mathcal {F}^{q-1}(\mathbb {C}). \end{aligned}$$

Pure polyanalytic spaces are important in signal analysis [2] and in connection to theoretical physics [5, 40]. Indeed, they parameterize the so-called *Landau levels*, which are the eigenspaces of the Landau Hamiltonian and model the distribution of electrons in high energy states (see e.g. [57, Section 2], [8, Section 4.1]).

The complex Hermite polynomials (1.4) provide a natural way of defining a polynomial subspace of the true polyanalytic space:

$$\begin{aligned} \mathcal {P}ol_{\pi ,r,N}=span\{H_{j,r}(z,\overline{z}):0\le j\le N-1\}. \end{aligned}$$

Thus,

$$\begin{aligned} Pol_{\pi ,q,N}=\mathcal {P}ol_{\pi ,0,N}\oplus \cdots \oplus \mathcal {P}ol_{\pi ,q-1,N}. \end{aligned}$$

The reproducing kernel of $Pol_{\pi ,r,N}$ is therefore

$$\begin{aligned} \mathcal {K}_{r,\pi ,N}(z,z^{\prime })=\sum _{j=0}^{N-1}H_{j,r}(z,\overline{z} ) \overline{H_{j,r}(z^{\prime },\overline{z^{\prime }})}, \end{aligned}$$

and the corresponding determinantal point processes have been introduced in [40].

## Footnotes

1 Perelomov [53] mentions that (1.4) has been used by Feynman and Schwinger as the explicit expression for the matrix elements of the displacement operator in Bargmann-Fock space.

2 The first Landau level is also called *ground level* because it corresponds to the lowest energy.

3 See also [51, Proposition 14] and [20], where it is pointed out that the sharp rate for the ground level also follows from pointwise estimates for Bergman kernels [60].

4 We do not denote this kernel by $K^g_{\Omega }$ in order to avoid a possible confusion with the restricted kernel ${K^g}_{|\Omega }$. Note also the notational difference between the finite ensemble $\mathcal {X}^g_\Omega$ and the restriction of the infinite ensemble ${\mathcal {X}^g}_{|\Omega }$.

5 The operator $H_{m}^{g}$ should not be confused with the complex Hermite polynomial $H_{j,r}$.
